# Exercise without Weight Loss Prevents Seasonal Decline in Vitamin D Metabolites: The VitaDEx Randomized Controlled Trial

**DOI:** 10.1002/advs.202416312

**Published:** 2025-05-11

**Authors:** Oliver J. Perkin, Sophie E. Davies, Martin Hewison, Kerry S. Jones, Javier T. Gonzalez, James A. Betts, Carl Jenkinson, Mark A. Lindsay, Sarah R. Meadows, Damon A. Parkington, Albert Koulman, Dylan Thompson

**Affiliations:** ^1^ Centre for Nutrition and Exercise Metabolism Department for Health University of Bath Bath BA2 7AY UK; ^2^ Institute of Metabolism and Systems Research University of Birmingham Birmingham B15 2TT UK; ^3^ Nutritional Biomarker Laboratory MRC Epidemiology Unit University of Cambridge Cambridge CB2 0SL UK; ^4^ University of Sydney Sydney New South Wales Australia; ^5^ MRC Laboratory of Medical Sciences Du Cane Road London W12 0HS UK; ^6^ Department of Life Sciences University of Bath Bath BA2 7AY UK

**Keywords:** 1,25(OH)D, Exercise, VitaDEx, Vitamin D, Weight Stable, Winter

## Abstract

Many adults become vitamin D deficient or insufficient during winter at northerly latitudes when cutaneous vitamin D synthesis does not occur. Vitamin D accumulates in adipose tissue and people with overweight or obesity are more likely to have low systemic vitamin D. This randomized controlled trial demonstrates that regular exercise completely maintains serum concentrations of the active vitamin D metabolite 1,25(OH)_2_D_3_ over winter and may ameliorate the decline in 25(OH)D status in overweight men and women, even without weight loss. The binding of 1,25(OH)_2_D_3_ to the vitamin D receptor mediates the crucial role for vitamin D in the healthy function of multiple organ systems and vitamin D supplementation does not impact circulating 1,25(OH)_2_D_3_. Thus, the VitaDEx study provides causal evidence that exercise plays an important role in vitamin D metabolism that is distinct from the effects of oral supplementation.

## Graphical abstract

In people with overweight or obesity, regular exercise even without weight loss or vitamin D supplementation completely prevents the usual winter decline in the vitamin D metabolite that maintains health at a cellular level, 1,25(OH)_2_D_3_, and ameliorates the decline in the vitamin D status marker 25(OH)D.

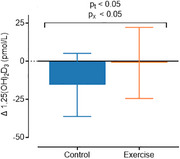

## 1 Introduction

Vitamin D is the umbrella term for the secosteroid hormones that support bone health^[^ ^1^ ^]^ and diverse physiological processes including immune function, metabolic health, and muscle function.^[^ ^2^ ^]^ Briefly, vitamin D_3_ (and D_2_) is hydroxylated in the liver into 25‐hydroxyvitamin D (25(OH)D), which is the precursor for the biologically active vitamin D metabolite 1,25(OH)2D which is produced in the kidneys in an endocrine fashion by the enzyme CYP27B1. Both 25(OH)D and 1,25(OH)2D can enter target cells, with the latter binding to the vitamin D receptor (VDR), to promote all actions of vitamin D. Target cells may also express the enzyme CYP27B1 to convert 25(OH)D to 1,25(OH)2D locally and promote intracrine VDR responses. Likewise, in both renal and extrarenal cells, 1,25(OH)D stimulates the enzyme CYP24A1, generating 24,25(OH)2D and promoting catabolism and excretion of vitamin D.^[^ ^3^ ^]^ The primary source of vitamin D in humans is cutaneous synthesis following exposure to solar ultraviolet radiation between 290 and 315 nm in wavelength, i.e., UVB in sunlight.^[^ ^4^ ^]^ For large parts of the year, insufficient UVB light reaches northern latitudes for cutaneous vitamin D synthesis to occur.^[^ ^5^ ^]^ As such, in countries such as the UK, 23% of the population become vitamin D deficient (serum 25(OH)D < 25 nmol L^−1^) throughout winter, and a further 48% become vitamin D insufficient (serum 25(OH)D < 50 nmol L^−1^).^[^ ^6^ ^]^ Regardless of season, obesity almost doubles the risk of vitamin D insufficiency and deficiency compared to healthy weight.^[^ ^6^ ^]^

As a lipophilic molecule, vitamin D accumulates in lipid droplets within adipocytes such that adipose tissue in humans contains large quantities of vitamin D.^[^ ^7^, ^8^ ^]^ Weight loss in nonsupplementing obese participants improves vitamin D status,^[^ ^9^ ^]^ and improvement in vitamin D status appears to be broadly proportional to the degree of weight loss.^[^ ^10^ ^]^ We previously proposed that improved vitamin D status may be directly explained by the release of vitamin D sequestered in adipose tissue into the circulation as a biproduct of lipid mobilization during weight loss.^[^ ^11^ ^]^ If this is the case, then lifestyle factors such as episodic exposure to lipolytic stimuli such as exercise or extended/intermittent fasting^[^ ^12^ ^]^ may also help to mobilize vitamin D from adipose tissue, potentially even without weight loss. This could be particularly relevant in the context of dysregulated adipose tissue function with greater adiposity,^[^ ^13^ ^]^ including adipose tissue insulin resistance and impaired adipose tissue responses to adrenergic stimulation,^[^ ^14^ ^]^ both of which are improved with exercise training.^[^ ^12^ ^]^

We recently reported that a single bout of exercise leads to transient increases in circulating 25(OH)D and 1,25(OH)_2_D_3_ in men and women.^[^ ^15^ ^]^ Exercise may be associated with longer lasting changes in circulating concentrations of vitamin D in humans; however, previous longitudinal studies have not controlled the confounding effects of weight loss, cutaneous vitamin D synthesis, or vitamin D supplementation.^[^ ^16^, ^17^ ^]^ There has been no longitudinal investigation on the effect of exercise on vitamin D status using gold‐standard analytical techniques (liquid chromatography‐tandem mass spectrometry [LC‐MS/MS]),^[^ ^18^, ^19^ ^]^ or rigorous study design controlling for UVB exposure.

In the VitaDEx randomized controlled trial (RCT), we investigated the effect of 10 weeks of regular exercise, without weight loss or vitamin D supplementation, on vitamin D status and metabolism in habitually sedentary people with overweight or obesity. In addition to the assessment of circulating 25(OH)D, we assessed other important vitamin D metabolites including active and catabolic forms (1,25(OH)_2_D_3_ and 24,25(OH)_2_D_3_, respectively).^[^ ^18^, ^19^, ^20^ ^]^ We also examined the effect of exercise on adipose tissue vitamin D concentrations, pathways involved in vitamin D metabolism, and whole‐body vitamin D expenditure rate using isotopic tracers.^[^ ^21^ ^]^ We hypothesized that exercise would attenuate the decline in serum 25(OH)D typically observed over winter in the UK.

## 2 Results

We conducted an RCT in individuals with overweight/obesity who were sedentary but otherwise free from known disease to examine changes in vitamin D concentrations over 10 weeks of winter (Exercise vs Control). The exercise group were prescribed four indoor cardiovascular exercise sessions per week for 10 weeks, while participants in the control group were asked to maintain their habitual lifestyle. Participants were randomized to groups by minimization^[^ ^22^ ^]^ based on age, sex, fat mass index (DXA fat mass in kg/height in m^2^; FMI), physical activity level (total energy expenditure/resting energy expenditure; PAL), and Fitzpatrick Skin Phototype.^[^ ^23^ ^]^ To isolate the effect of exercise on vitamin D status, all outcome measurements and intervention activities were scheduled to take place between October 01 and April 01 from 2019 to 2022 to ensure no cutaneous vitamin D synthesis occurred, owing to the lack of 290–315 nm radiation in sunlight at northerly latitudes during winter.^[^ ^5^ ^]^ Polysulfone badges were not used to record participant sunlight exposure owing to their sensitivity to wavelength of light up to 330 nm, and the potential for inaccurate placement over a 10‐week intervention.^[^ ^24^ ^]^ Furthermore, all participants were required to refrain from vitamin D supplementation and maintain total body mass over the intervention, with the exercise group increasing daily energy intake to match additional energy expenditure due to exercise.

The primary outcome measure was change in serum total 25(OH)D concentration (25(OH)D_2_ and 25(OH)D_3_) from baseline‐to‐post‐intervention measured by LC‐MS/MS. Secondary outcome measures included baseline‐to‐post‐intervention changes in serum concentrations of 25(OH)D_3_, 25(OH)D_2_, 1,25(OH)_2_D_3_, 24,25(OH)_2_D_3_, 3‐Epi‐25(OH)D_3_, and vitamin D_3_ (cholecalciferol), and subcutaneous adipose tissue concentrations of 25(OH)D and vitamin D_3_, all measured by LC‐MS/MS. Serum vitamin D binding protein (DBP) and albumin were assessed using enzyme‐linked immunosorbent assay (ELISA). As a biomarker for whole body 25(OH)D_3_ expenditure, half‐life of 25(OH)D_3_ was calculated from the disappearance rate of stable isotope tracer (d_3_‐25(OH)D_3_) in plasma measured with LC‐MS/MS. Free and bioavailable 25(OH)D concentrations,^[^ ^25^ ^]^ and vitamin D metabolite ratios (25(OH)D_3_:24,25(OH)_2_D_3_, and 1,25(OH)_2_D_3_:24,25(OH)_2_D_3_) were calculated.^[^ ^20^ ^]^ The change in the expression of vitamin D metabolism‐related genes in subcutaneous adipose was determined using RNA‐Seq. Biomarkers associated with vitamin D metabolism (total calcium, parathyroid hormone [PTH], DBP, and albumin), liver function (AST and ALT), metabolic health (fasted glucose, insulin, TAG, NEFA, leptin, and HDL and LDL cholesterol), and systemic inflammation (IL‐6 and CRP) were also measured. Body composition was measured with dual‐energy X‐ray absorptiometry (DXA), and tibial bone and calf muscle characteristics were measured with peripheral quantitative computed tomography (pQCT). Data were analyzed with linear mixed effects modeling (LMEM) where the effect of time was considered an important contextual finding, or with ANCOVA with baseline as a covariate for the remaining outcomes. No obvious deviations from normality of residuals were observed, such that no adjustments to analysis were required given the sample size.^[^ ^26^ ^]^

### 2.1 Participants

**Figure 1:**
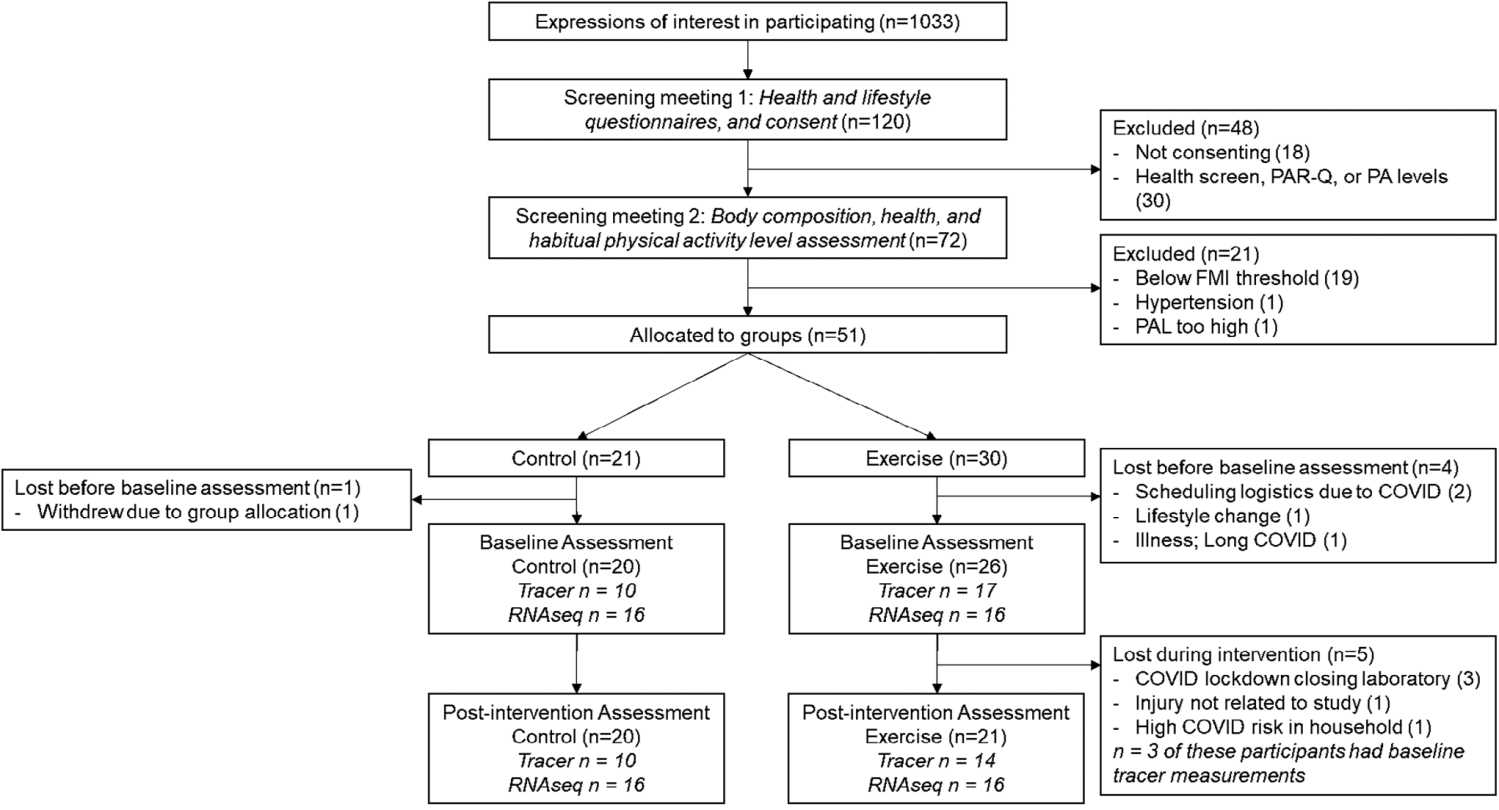
CONSORT flow diagram. FMI; fat mass index, PA; physical activity, PAL; physical activity level as a ratio of total to resting energy expenditure, PAR‐Q; Physical Activity Readiness Questionnaire.

Fifty‐one participants passed screening based on eligibility criteria listed in the Experimental Section and were allocated to groups by a statistician external to the research. Initially, group allocation was on a 1:1 basis in the first winter of testing. Due to the impact of COVID‐19 lockdown restrictions, which disproportionately increased participant withdrawal from the exercise group, the allocation ratio was altered to 2:1 in the second and third winters of the study to ensure sufficient sample size was achieved in both groups. Twenty‐one participants were allocated to the control group, with *n* = 20 completing, and 30 participants allocated to the exercise group with *n* = 21 completing (*n* = 7 lost to a combination of COVID closures, illness, or risk) (**Figure** **1**).

Baseline participant characteristics at screening are displayed in **Table** **1**.

**Table 1:** Baseline characteristics at study group allocation.

	Control (*n* = 20)	Exercise (*n* = 21)
Females *n* (%)	12 (60%)	11 (52%)
Age (years)	49 ± 12	49 ± 11
Body mass (kg)	98.3 ± 11.4	98.6 ± 15.1
BMI (kg m^−2^)	34.3 ± 4.0	33.6 ± 4.2
Percentage body fat (%)	41.0 ± 7.1	39.2 ± 8.0
FMI (fat mass kg m^−2^)	13.9 ± 3.5	14.2 ± 3.8
PAL (TEE/REE)	1.63 ± 0.17	1.69 ± 0.19
MVPA min/day (≥3<10.2 METs)	75 ± 48	101 ± 58
Fitzpatrick skin type total 40	18 ± 4	17 ± 5
Fitzpatrick skin type 1 *n* (%)	0 (0%)	1 (5%)
Fitzpatrick skin type 2 *n* (%)	10 (50%)	11 (52%)
Fitzpatrick skin type 3 *n* (%)	9 (45%)	9 (43%)
Fitzpatrick skin type 4 *n* (%)	1 (5%)	0 (0%)

Data presented as mean ± SD unless stated. Abbreviations: BMI, body mass index; FMI, fat mass index from dual‐energy X‐ray absorptiometry; PAL, physical activity level as total energy expenditure/resting energy expenditure; MVPA, moderate‐to‐vigorous physical activity between 3 and 10.2 metabolic equivalents.

### 2.2 Exercise Ameliorates the Seasonal Decline in Vitamin D Status

**Figure 2:**
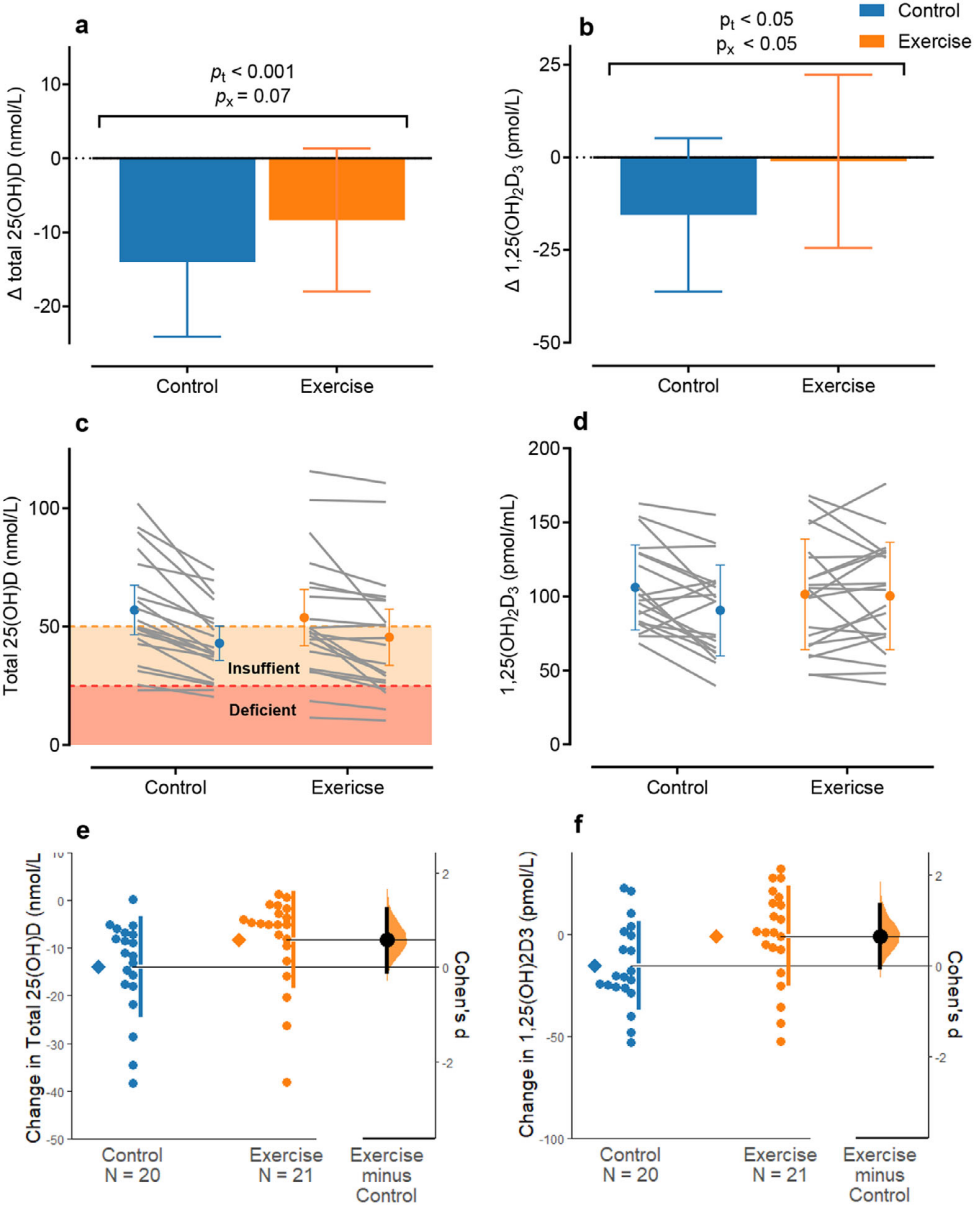
Responses of a,c,e) serum total 25(OH)D and b,d,f) 1,25(OH)_2_D_3_ with the control group in blue and exercise group in orange throughout. The mean ± SD change over 10 weeks of winter for serum concentrations of total 25(OH)D and 1,25(OH)_2_D_3_ are displayed in (a) and (b), respectively. Individual participant changes from baseline‐to‐post intervention, with mean [95% CI], are displayed in (c) and (d), with cut‐offs for vitamin D insufficiency and deficiency based on serum 25(OH)D are demarcated in (c). Cohen's *d* effect size [bootstrapped 95% CI]^[^ ^27^ ^]^ is presented in (e) and (f) for the difference in baseline‐to‐post‐intervention change in total 25(OH)D concentration between groups with the control group in blue and exercise group in orange and means represented as *p*_x_ = *p* value for interaction effects, and *p*_t_ = *p* value for the main effect of time, comparing the change over time between groups with LMEM.

Serum concentrations of the active vitamin D metabolite 1,25(OH)_2_D_3_ were completely preserved in the exercise group (**Figure** **2**b). The difference in decline in total 25(OH)D showed evidence for amelioration with exercise that was not statistically significant (Figure 2a). The group × time interaction effect (*p_x_*) for total 25(OH)D was *p_x_* = 0.07, with a mean difference in concentration change between groups of 5.7 nmol L^−1^ [95% CI −0.2 to 11.5 nmol L^−1^], and a moderate effect size for the difference in concentration change between groups *d* = 0.58 [95% CI −0.13 to 1.21]) (Figure 2a,c,e). In the exercise group, 1,25(OH)_2_D_3_ concentration was maintained over winter, compared to a 15% decline in the control group (*p_x_* = 0.04, *p_t_ =* 0.02). The mean difference in the concentration change between groups was 14.4 pmol L^−1^ [95% CI 0.5–26.8 pmol L^−1^], with a moderate effect size of *d* = 0.65 [95% CI 0.00–1.37] (Figure 2b,d,f).

**Figure 3:**
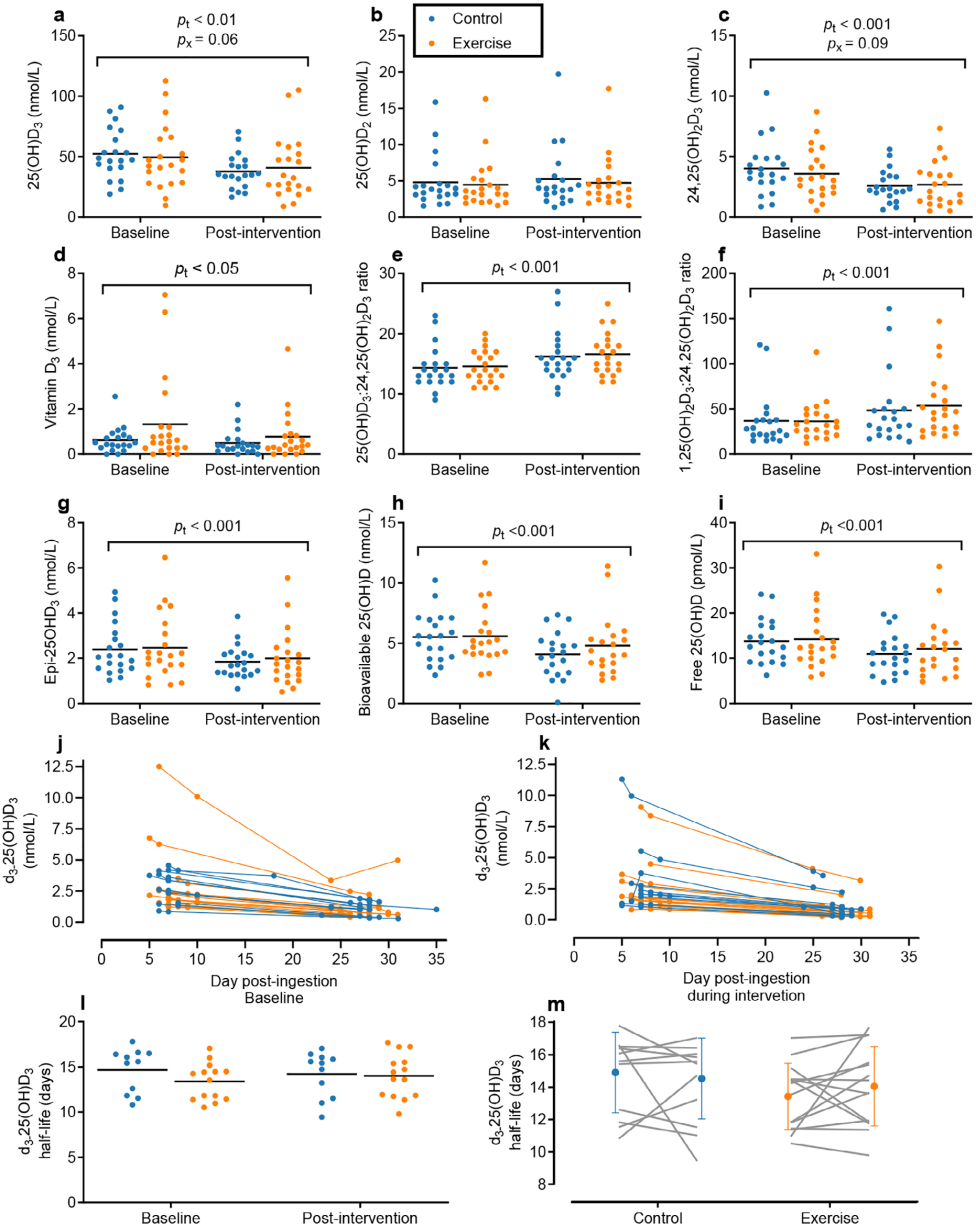
Responses of serum vitamin D metabolites, ratios, and half‐life. Mean with individual data points are presented for serum a) 25(OH)D_3_, b) 25(OH)D_2_, c) 24,25(OH)_2_D_3_, d) vitamin D_3_, e) vitamin D metabolite ratios 25(OH)D_3_:24,25(OH)_2_D_3_, and f) 1,25(OH)_2_D_3_:24,25(OH)_2_D_3_, g) serum Epi‐25(OH)D_3_, and calculated bioavailable h) 25(OH)D and i) free 25(OH)D. Individual (Control *n* = 11, Exercise *n* = 14) time course responses over the four weeks to ingestion of 60 nmol of labeled d_3_‐25(OH)D_3_ j) immediately prior to the intervention or k) during the last four weeks of the intervention from which the d_3_‐25(OH)D_3_ half‐life was estimate by linear regression. l,m) Mean with individual data points, and individual participant responses from baseline‐to‐post‐intervention are displayed for d_3_‐25(OH)D_3_ tracer half‐life control group in blue and exercise group in orange. Free and bioavailable 25(OH)D data were only available for *n* = 20 for the Exercise group due to missing albumin data for one participant. *p*_x_ = *p* value for interaction effects, and *p*_t_ = *p* value for the main effect of time, comparing the change over time between groups with LMEM.

Serum 25(OH)D_3_ displayed the same response as total 25(OH)D, with a significant time effect (*p_t_ <* 0.01) and an interaction effect of *p_x_* = 0.06, with a mean difference in concentration change of 5.9 nmol L^−1^ [95% CI 0.2–11.6 nmol L^−1^] and an effect size for the difference in concentration change of *d* = 0.60 [95% CI −0.12 to 1.24] (**Figure** **3**a). There was no time or interaction effect for serum 25(OH)D_2_ concentrations (Figure 3b). Serum 24,25(OH)_2_D_3_ declined significantly during the intervention period (*p_t_* < 0.001), with an interaction effect of *p_x_* = 0.09. The mean difference in concentration change was 0.5 nmol L^−1^ [95% CI 0.0–1.1] with a moderate effect size (*d* = 0.53 [95% CI −0.22 to 1.08]) between groups (Figure 3c).

### 2.3 Exercise Does Not Alter the Seasonal Response of Markers of Vitamin D Metabolism and Turnover over Winter

Significant declines were observed in serum vitamin D_3_ (*p_t_* < 0.05), 3‐Epi‐25(OH)D_3_ (*p_t_* < 0.001), bioavailable 25(OH)D (*p_t_* < 0.001), and free 25(OH)D (*p_t_* < 0.001) in both groups over the 10‐week intervention, with no differences in the extent of decline between groups (see Figure 3d,g,h,i, respectively). Vitamin D metabolite ratios (25(OH)D_3_:24,25(OH)_2_D_3_ and 1,25(OH)_2_D_3_:24,25(OH)_2_D_3_) increased over the winter in both groups (both *p_t_* < 0.0001), with no group × time interaction effects observed (*p_x_* = 0.51 and *p_x_* = 0.25, respectively; Figure 3e,f).

In the subgroup of participants completing assessment of vitamin D expenditure (control group *n* = 11, exercise group *n* = 14), there were no changes in d_3_‐25(OH)D_3_ half‐life in plasma over the intervention in either group (*p_t_* = 0.86 and *p_x_* = 0.32; Figure 3j–m). Over the four weeks before the intervention, 25(OH)D_3_ half‐life was 14.7 ± 2.5 and 13.4 ± 2.0 d in the control and exercise groups, respectively. During the final four weeks of the intervention, half‐life was 14.2 ± 2.5 and 14.0 ± 2.5 d in the control and exercise groups, respectively.

### 2.4 Concentrations of Vitamin D in Subcutaneous Adipose Tissue Decrease over the Winter

**Figure 4:**
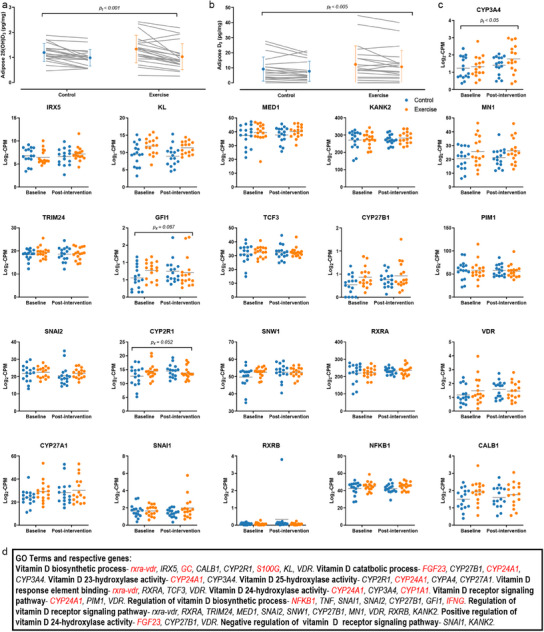
Adipose tissue vitamin D concentration and gene expression. Mean ± SD is presented with individual participant responses from baseline‐to‐post‐intervention for a) adipose tissue 25(OH)D and b) vitamin D_3_ concentrations with control group in blue and exercise group in orange. c) The expression of adipose tissue genes involved in vitamin D metabolism is presented as mean ± SD at baseline and post‐intervention from *n* = 16 control and *n* = 16 exercise participants, d) as identified based on the GO Terms listed. Genes in red text were not expressed in adipose. *p*_x_ = *p* value for interaction effects, and *p*_t_ = *p* value for the main effect of time, comparing the change over time between groups with LMEM.

In both groups, adipose concentrations of 25(OH)D_3_ and vitamin D_3_ decreased significantly over 10 weeks of winter (*p_t_* <0.001 and *p_t_* < 0.005, respectively). However, there were no differences in the magnitude of decrease between groups. Adipose 25(OH)D_3_ concentration decreased by 0.26 ± 0.24 and 0.30 ± 0.31 pg mg^−1^ in the control and exercise groups, respectively, with an effect size for difference in change scores of *d* = 0.35 [95.0% CI −0.33 to 0.93]. Adipose vitamin D_3_ concentration decreased by 1.46 ± 3.15 and 1.63 ± 3.31 pg mg^−1^, in the control and exercise groups, respectively (*d* = 0.17 [95.0% CI −0.48 to 0.81]) (**Figure** **4**a,b).

In a subset of participants (control *n* = 16, exercise *n* = 16), analysis of the expression of individual genes involved vitamin D metabolic pathways in adipose tissue found that, broadly, gene expression was unchanged when comparing the difference in the log_2_‐CPM from baseline‐to‐post‐intervention with a LMEM (Figure 4c). The AmiGO web‐based gene ontology toolset was used to identify genes involved in vitamin D metabolism (see the Experimental Section for pathway GO terms included). Of note, there was a significant main effect of time (*p_t_* < 0.05) for *CYP3A4* expression, but no interaction effect with LMEM, and interaction effects for *CYP2R1* and *GFI1* expression were *p_x_* = 0.052 and *p_x_* = 0.057, respectively. Also of note, two key vitamin D related genes, *GC* (a vitamin D binding protein gene) and *CYP24A1* (vitamin D_3_ 24‐hydroxylase) did not appear to be expressed in adipose tissue (Figure 4d).

### 2.5 The Intervention Was Well‐Adhered to and Effective at Improving Physical Activity and Fitness

Exercise intervention adherence was 88%, with participants completing 35 ± 5 of 40 sessions over the 10‐week intervention period. All but two participants completed 30 or more exercise sessions, with the two exceptions each completing 24 sessions. Removing these two participants from the analysis did not alter any outcomes. The individualized training programs prescribed 166 ± 16 min of exercise per week. The exercise intervention resulted in significant improvements in V̇O_2_max, in absolute terms and relative to body mass, and significant increases in maximal rate of fat oxidation compared to the control group (**Table** **2**). Participants in the exercise group decreased daily sedentary time (<1.5 METs) and increased MVPA time (≥3<10.2 METs), significantly increasing PAL with an estimated mean increase in total daily energy expenditure of 305 kcal [95% CI 164–447 kcal] compared to pre‐intervention (Table 2). All metrics of physical activity remained stable in the control group.

**Table 2:** Baseline to post‐intervention changes in confirmatory outcomes.

Measurement	Group	*n*	Baseline	Post‐intervention	Within group estimated mean difference	[95% CI]	Between group estimated mean difference	[95% CI]	GLM*p‐*value	Bootstrapped effect size of change scores	
			Mean ± SD	Mean ± SD						*d*	[95% CI]
Body mass (kg)	Con	20	98.3 ± 11.4	98.9 ± 11.3	0.6	*−0.2 to 1.5*	0.1	*−1.1 to 1.2*	0.91	0.03	*−0.66 to 0.61*
	Ex	21	98.6 ± 15.1	99.3 ± 15.5	0.7	*−0.1 to 1.5*					
BMI (kg m^−2^)	Con	20	34.3 ± 4.0	34.5 ± 3.9	0.2	*−0.1 to 0.5*	0.0	*−0.4 to 0.4*	0.87	0.04	*−0.66 to 0.61*
	Ex	21	33.6 ± 4.2	33.8 ± 4.4	0.3	*−0.0 to 0.5*					
% body fat (%)	Con	20	41.0 ± 7.1	41.7 ± 7.4	0.6	*−0.1 to 1.3*	−0.5	*−1.4 to 0.5*	0.33	0.36	*−0.29 to 0.96*
	Ex	21	39.2 ± 8.0	39.3 ± 8.5	0.1	*−0.6 to 0.8*					
Total fat mass (kg)	Con	20	39.4 ± 7.6	40.3 ± 7.9	0.8	−0.6 to 1.7	−0.4	*−1.7 to 0.8*	0.48	0.25	*−0.40 to 0.86*
	Ex	21	38.2 ± 10.5	38.6 ± 11.3	0.4	−0.5 to 1.2					
FMI (kg m^−2^)	Con	20	13.9 ± 3.5	14.2 ± 3.6	0.3	−0.0 to 0.6	−0.1	*−0.6 to 0.3*	0.58	0.11	*−0.55 to 0.76*
	Ex	21	14.2 ± 3.8	13.2 ± 4.1	0.2	−0.1 to 0.5					
FFM (kg)	Con	20	57.0 ± 10.1	56.7 ± 10.3	−0.3	−0.9 to 0.4	0.5	*−0.4 to 1.5*	0.26	0.38	*−0.29 to 1.02*
	Ex	21	58.6 ± 10.1	58.8 ± 10.5	0.3	−0.4 to 0.9					
33% tibia BMD (g cm^−2^)	Con	20	1.13 ± 0.28	1.13 ± 0.28	0.00	−0.05 to 0.06	−0.01	*−0.08 to 0.07*	0.89	0.04	*−0.43 to 0.87*
	Ex	21	1.12 ± 0.33	1.12 ± 0.33	0.00	−0.05 to 0.05					
Calf mCSA (cm^2^)	Con	20	85.4 ± 17.3	86.1 ± 18.2	0.7	−0.3 to 1.7	−0.3	*−1.7 to 1.1*	0.66	0.09	*−0.55 to 0.73*
	Ex	21	88.9 ± 14.4	89.4 ± 14.9	0.4	−0.5 to 1.4					
Calf muscle density (mg cm^−2^)	Con	20	75.6 ± 3.0	75.1 ± 2.8	−0.5	−1.2 to 0.1	0.7	*−0.2 to 1.6*	0.11	0.47	*−0.24 to 1.05*
	Ex	21	75.9 ± 1.3	76.1 ± 2.0	0.2	−0.4 to 0.8					
Serum DBP (mg dL^−1^)	Con	20	338 ± 63	329 ± 45	−5	−21 to 12	−5	*−29 to 18*	0.66	0.07	*−0.59 to 0.72*
	Ex	21	322 ± 43	316 ± 43	−10	−16 to 7					
Plasma iPTH (pmol L^−1^)	Con	20	4.0 ± 1.5	4.6 ± 1.6	0.6	0.2–0.9	−0.2	*−0.7 to 0.3*	0.41	0.21	*−0.85 to 0.43*
	Ex	20^a^	3.5 ± 1.1	3.8 ± 1.3	0.4	0.0–0.7					
Plasma albumin (g L^−1^)	Con	20	44.0 ± 3.5	43.7 ± 2.6	−0.3	−1.4 to 0.7	0.2	*−1.3 to 1.7*	0.74	0.02	*−0.63 to 0.67*
	Ex	20^a^	44.5 ± 3.6	44.2 ± 3.4	−0.1	−1.1 to 1.0					
Serum total calcium (mmol L^−1^)	Con	17^a^	2.27 ± 0.08	2.27 ± 0.09	0.00	−0.03 to 0.03	0.01	*−0.03 to 0.05*	0.59	0.22	*−0.50 to 0.92*
	Ex	19^a^	2.28 ± 0.05	2.27 ± 0.06	0.01	−0.02 to 0.04					
AST (IU L^−1^)	Con	20	23 ± 10	25 ± 12	2	−1 to 5	−2	*−6 to 2*	0.28	0.31	*−0.38 to 0.87*
	Ex	21	20 ± 6	21 ± 6	1	−3 to 3					
ALT (IU L^−1^)	Con	20	24 ± 7	25 ± 7	1	0–3	0	*−3 to 2*	0.91	0.00	*−0.65 to 0.64*
	Ex	21	23 ± 7	24 ± 6	1	0–3					
Serum IL‐6 (pg mL^−1^)	Con	19^a^	1.27 ± 0.67	1.20 ± 0.61	−0.06	−0.30 to 0.18	−0.04	*−0.37 to 0.30*	0.82	0.03	*−0.59 to 0.69*
	Ex	21	1.08 ± 0.39	1.18 ± 0.48	−0.10	−0.33 to 0.13					
Serum CRP (mg L^−1^)	Con	19^a^	5.1 ± 3.6	4.6 ± 3.6	−0.5	−2.0 to 1.1	0.4	*−1.8 to 2.6*	0.71	0.16	*−0.54 to 0.73*
	Ex	21	3.1 ± 2.5	3.1 ± 2.9	−0.1	−1.6 to 1.4					
Serum insulin (pmol L^−1^)	Con	19^a^	49 ± 21	51 ± 27	−1	−11 to 9	5	*−10 to 19*	0.51	0.04	*−0.63 to 0.70*
	Ex	21	71 ± 32	72 ± 31	4	−6 to 13					
Serum glucose (mmol L^−1^)	Con	20	5.3 ± 0.7	5.5 ± 0.9	0.9	−0.2 to 0.4	−0.1	*−0.5 to 0.4*	0.83	0.23	*−0.44 to 0.87*
	Ex	19^a^	5.8 ± 0.7	5.7 ± 0.8	0.4	−0.3 to 0.4					
Serum leptin (ng mL^−1^)	Con	18^a^	46.8 ± 30.1	49.4 ± 36.5	1.0	−4.0 to 6.0	−1.3	*−8.2 to 5.6*	0.70	0.19	*−0.47 to 0.76*
	Ex	21	39.2 ± 21.3	39.8 ± 23.7	−0.3	−5.0 to 4.3					
Serum TAG (mmol L^−1^)	Con	20	1.1 ± 0.7	1.1 ± 0.5	−0.1	−0.3 to 0.1	0.3	*0.0–0.5*	**0.03**	0.42	*−0.19 to 0.96*
	Ex	21	1.3 ± 0.7	1.5 ± 0.6	0.2	0.0–0.3					
Serum NEFA (mmol L^−1^)	Con	19^a^	0.5 ± 0.1	0.5 ± 0.2	0.0	−0.1 to 0.1	−0.3	*−0.2 to 0.1*	0.73	0.04	*−0.58 to 0.70*
	Ex	21	0.5 ± 0.2	0.4 ± 0.2	0.0	−0.1 to 0.1					
Serum LDL (mmol L^−1^)	Con	20	3.0 ± 0.7	2.9 ± 0.6	−0.2	−0.4 to 0.1	0.0	*−0.3 to 0.3*	0.77	0.21	*−0.43 to 0.84*
	Ex	21	3.6 ± 0.9	3.8 ± 0.8	−0.2	−0.4 to 0.0					
Serum HDL (mmol L^−1^)	Con	20	1.4 ± 0.3	1.4 ± 0.3	0.0	−0.1 to 0.1	0.0	*−0.1 to 0.0*	0.32	0.27	*−0.34 to 0.89*
	Ex	21	1.3 ± 0.3	1.3 ± 0.4	0.0	−0.1 to 0.0					
Total cholesterol (mmol L^−1^)	Con	20	4.6 ± 0.8	4.5 ± 0.6	−0.1	−0.4 to 0.2	−0.2	*−0.6 to 0.3*	0.45	0.27	*−0.35 to 0.89*
	Ex	21	5.1 ± 0.9	5.4 ± 1.0	−0.3	−0.6 to 0.0					
Adipo IR (pmol L^−1^ × nmol L^−1^)	Con	18^a^	23.3 ± 10.7	23.7 ± 10.2	−1.5	−7.9 to 8.7	0.4	*−8.7 to 9.4*	0.94	0.16	*−0.57 to 0.76*
	Ex	20^a^	30.2 ± 13.8	33.0 ± 25.9	−1.1	−8.7 to 9.4					
V̇O_2_max (mL kg^−1^ min^−1^)	Con	20	23.2 ± 3.4	23.5 ± 4.0	0.1	−1.0 to 1.1	1.8	*0.2–3.3*	**0.03**	0.57	*−0.21 to 1.26*
	Ex	19^b^	27.3 ± 6.9	28.9 ± 6.3	1.8	0.8–2.9					
V̇O_2_max (L min^−1^)	Con	20	2.3 ± 0.4	2.3 ± 0.5	0.0	−0.1 to 0.1	0.2	*0.0–0.3*	**0.03**	0.60	*−0.18 to 1.33*
	Ex	19^b^	2.7 ± 0.7	2.9 ± 0.6	0.2	0.1–0.3					
MFO (g min^−1^)	Con	20	0.33 ± 0.10	0.30 ± 0.10	−0.03	−0.08 to 0.02	0.08	*0.01–0.15*	**0.02**	0.66	*−0.01 to 1.25*
	Ex	19^c^	0.33 ± 0.11	0.38 ± 0.13	0.05	0.00–0.10					
Vitamin D intake (µg d^−1^)	Con	16^d^	3.6 ± 3.5	2.8 ± 2.6	−0.1	−1.1 to 0.8	−0.3	*−1.6 to 1.0*	0.64	0.26	*−0.50 to 0.87*
	Ex	21	1.5 ± 1.0	1.6 ± 1.5	−0.4	−1.2 to 0.4					
PAL (TEE/REE)	Con	18^e^	1.63 ± 0.17	1.61 ± 0.16	−0.03	−0.12 to 0.05	0.21	*0.10–0.33*	**<0.001**	1.00	*0.33–1.53*
	Ex	20^e^	1.69 ± 0.19	1.86 ± 0.26	0.18	0.10–0.26					
Total daily EE (kcal)	Con	18^e^	2965 ± 602	2992 ± 619	−5	−154 to 144	310	*104–516*	**<0.005**	0.86	*0.17–1.46*
	Ex	20^e^	3074 ± 705	3379 ± 800	305	164–447					
Sed min/day (<1.5 METs)	Con	18^e^	560 ± 108	589 ± 120	29	−22 to 80	−105	*−176 to 35*	**<0.005**	0.71	*0.04–1.30*
	Ex	20^e^	584 ± 115	504 ± 141	−76	−124 to −28					
MVPA min d^−1^ (≥3<10.2 METs)	Con	18^e^	75 ± 48	71 ± 48	−8	−34 to 17	53	*17–89*	**<0.005**	0.98	*0.26–1.46*
	Ex	20^e^	101 ± 58	145 ± 87	45	20–69					

Observed data at baseline and after the intervention for *n* participants in each group, presented as mean ± SD. The mean differences presented are baseline to post‐intervention within group with 95% confidence intervals [95% CI] and between group differences in change scores as Exercise minus Control with 95% confidence intervals [95% CI]; *p*‐values are presented for differences in change scores compared by GLM with baseline as a covariate both within groups and between groups; and effect sizes are the difference between baseline‐to‐post‐intervention change scores between groups, where 5000 bootstrap samples were taken and confidence intervals bias‐corrected and accelerated.^[^ ^27^ ^]^ Abbreviations: BMI, body mass index; FMI, fat mass index from dual‐energy X‐ray absorptiometry; FFM, fat free mass; BMD, bone mineral density; mCSA, muscle cross sectional area; iPTH, intact parathyroid hormone; AST, aspartate transferase; ALT, alanine transaminase; DBP, vitamin D binding protein; IL‐6, interleukin‐6; CRP, C‐reactive protein; TAG, triglyceride; NEFA, non‐esterified fatty acids; HDL, high‐density lipoprotein cholesterol, LDL, low‐density lipoprotein cholesterol; Adipo IR, adipose tissue insulin resistance index; V̇O_2_max, maximal rate of oxygen consumption; MFO, maximal rate of fat oxidation; PAL, physical activity level as total energy expenditure (TEE)/resting energy expenditure (REE); EE, energy expenditure; Sed, sedentary activity below 1.5 metabolic equivalents; MVPA, moderate‐to‐vigorous physical activity between 3 and 10.2 metabolic equivalents. Reasons for reduced sample size: ^a^reduced *n* due to insufficient supernatant at post‐intervention timepoint for all analysis for all participants; ^b^valid V̇O_2_max not attained based on criteria outlined in STAR methods; ^c^technical error during Douglas bag analysis; ^d^3‐d food diary during intervention not returned; ^e^insufficient wear time (<5 complete days) of Actiheart 5 monitor achieved during intervention physical activity monitoring period.

Total body mass was maintained in both study groups as required in the study design, and no participants lost ≥2% body mass between laboratory visits (see Figure 6 for schedule of laboratory visits). There were no significant differences between groups in the changes of any markers of whole‐body composition. There were also no changes in tibial bone mineral density or calf muscle CSA or density between or within groups across the intervention period (Table 2).

There were no differences in change in self‐reported daily vitamin D intake between groups, and no difference in change in DBP, PTH, total calcium, albumin concentrations, or AST and ALT activity. Apart from serum TAG, which decreased in the control group and increased in the intervention group (−0.1 mmol L^−1^ [95% CI −0.3 to 0.1 mmol L^−1^] vs 0.2 mmol L^−1^ [95% CI 0.0–0.3 mmo L^−1^], respectively), fasted serum metabolic and inflammatory markers did change in response to the intervention compared to control (Table 2).

### 2.6 Exercise Alters the Relationship between the Change in Serum 25(OH)D and Vitamin D_3_ Metabolites

**Figure 5:**
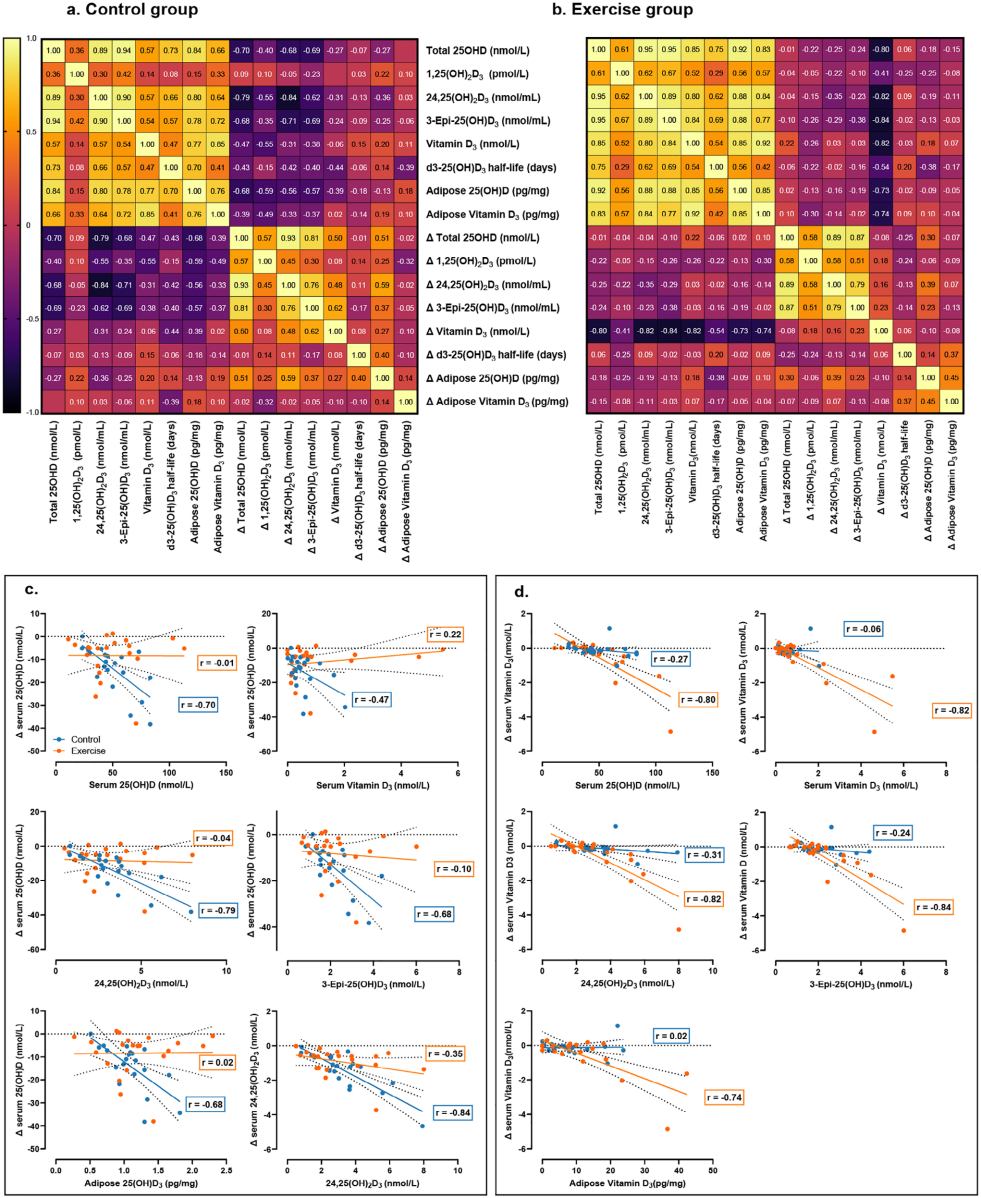
Exploratory analysis of relationships between concentration and change in vitamin D metabolites. Heat maps displaying the strength of Pearson's correlations as *r* values for concentrations and change scores (Δ) of vitamin D metabolite concentrations measured in serum and adipose tissue for the a) control group and b) exercise groups calculated using the Oldham method.^[^ ^28^ ^]^ Group level correlations (control group in blue and exercise group in orange) are displayed for correlations that differed between groups according to Fisher's *z* and Zou's 95% confidence intervals^[^ ^30^ ^]^ in (c) for change in serum 25(OH)D, with total serum 25(OH)D, serum vitamin D_3_, 24,25(OH)_2_D_3,_ 3‐Epi‐25(OH)D_3_, and adipose 25(OH)D_3_, and also change in 24,25(OH)_2_D_3_ with 24,25(OH)_2_D_3_, i.e., instance where there was a stronger negative correlation in the control group, and in (d) for change in serum vitamin D_3_ with total serum 25(OH)D, serum vitamin D_3_, 24,25(OH)_2_D_3,_ 3‐Epi‐25(OH)D_3_, and adipose D_3_, i.e., where there were stronger negative correlations in the exercise group compared to the control group.

Exploratory analysis of relationships between vitamin D metabolites in serum and adipose revealed divergent responses between groups for key vitamin D metabolites. The Oldham method was used to examine these relationships, taking the mean of baseline and post‐intervention instead of baseline only to avoid the effect of regression to the mean and reduce the effect of mathematical coupling.^[^ ^28^, ^29^ ^]^ Correlation matrices for control and exercise groups are displayed in **Figure** **5**a,b, respectively. The differences in correlations between groups was compared with Fisher's *z* and Zou's 95% confidence intervals.^[^ ^30^ ^]^ Correlations that differed between groups are described herein and presented in **Table** **3**.

**Table 3:** Divergent correlations between change in serum 25(OH)D or vitamin D_3_ and concentration of vitamin D metabolites.

Correlations between metabolite concentrations and serum 25(OH)D									
Metabolite	Control			Exercise			Between group difference		
	*r*	*[95% CI]*	*p*−value	*r*	*[95% CI]*	*p*‐value	Fisher's *z*	*[95% CI]*	*p‐*value
Serum 25(OH)D	**−0.70**	***−0.87 to −0.37***	**<0.001**	−0.01	*−0.44 to 0.43*	0.98	−2.54	*−1.16 to −0.15*	<0.05
Serum vitamin D_3_	**−0.47**	***−0.76 to −0.03***	**<0.05**	0.22	*−0.23 to 0.59*	0.34	−2.17	−1.16 to −0.06	<0.05
24,25(OH)_2_D_3_	**−0.79**	***−0.91 to −0.54***	**<0.001**	−0.04	*−0.47 to 0.39*	0.85	−3.08	−1.10 to −0.21	<0.01
3‐Epi‐25(OH)D_3_	**−0.68**	***−0.86 to −0.34***	**<0.05**	−0.10	*−0.51 to 0.35*	0.68	−2.16	−1.06 to −0.05	<0.05
Adipose 25(OH)D	**−0.68**	***−0.86 to −0.33***	**<0.0001**	0.02	*−0.42 to 0.45*	0.94	−2.51	−1.17 to −0.15	<0.05
Correlations between metabolite concentrations and serum vitamin D_3_									
Serum 25(OH)D	−0.27	*−0.64 to 0.19*	0.25	**−0.80**	***−0.92 to −0.57***	**<0.0001**	2.43	0.09–1.01	<0.05
Serum vitamin D_3_	−0.06	*−0.49 to 0.40*	0.81	**−0.82**	***−0.92 to −0.59***	**<0.001**	3.24	0.27–1.22	<0.01
24,25(OH)_2_D_3_	−0.31	*−0.66 to 0.15*	0.18	**−0.82**	***−0.92 to −0.60***	**<0.0001**	2.47	0.10–0.99	<0.05
3‐Epi‐25(OH)D_3_	−0.24	*−0.62 to 0.23*	0.31	**−0.84**	***−0.93 to −0.63***	**<0.0001**	2.88	0.17–1.08	<0.01
Adipose vitamin D_3_	0.02	*−0.42 to 0.46*	0.92	**−0.74**	***−0.89 to −0.46***	**<0.001**	2.87	0.23–1.22	<0.01

In the control group, the change in serum 25(OH)D over winter was strongly negatively correlated with concentrations of both serum and adipose 25(OH)D, as well as serum vitamin D_3_, 24,25(OH)_2_D_3_, and 3‐Epi‐25(OH)D_3_ (Figure 5c and Table 3). In the exercise group, these correlations did not exist. Conversely, the change in serum vitamin D_3_ was strongly negatively correlated with concentrations of serum 25(OH)D, vitamin D_3_, 24,25(OH)_2_D_3_, and 3‐Epi‐25(OH)D_3_, and adipose 25(OH)D, with these correlations not being present in the control group (Figure 5d and Table 3). There was also a difference in correlations between groups in concentration of 24,25(OH)_2_D_3_ and change in 24,25(OH)_2_D_3_ concentration (control *r* = −0.84 [95% CI −0.94 to −0.64], *p* < 0.0001; vs exercise *r* = −0.34 [95% CI −0.68 to 0.09], *p* = 0.12; between group difference *z* = −2.53 [95% CI −0.95 to −0.10], *p* = 0.01; Figure 5c). Removing the two outliers from the exercise group with concentrations of serum and adipose vitamin D_3_ and change in serum and adipose vitamin D_3_ more than 2 standard deviations above the respective means did not alter correlations or between group differences in correlations.

Of note, when combining groups, serum 25(OH)D concentration was positively correlated with d_3_‐25(OH)D_3_ half‐life at baseline (*r* = 0.64 [95% CI 0.32–0.83], *p* < 0.001) (Figure 5a,b).

## 3 Discussion

The VitaDEx study provides the first evidence using gold‐standard analytical techniques within a tightly controlled RCT that regular exercise may be a strategy to mitigate seasonal declines in circulating 25(OH)D and 1,25(OH)_2_D_3_ concentration during winter, even without vitamin D supplementation or weight loss. In people with overweight/obesity who undertook 10 weeks of exercise over the winter, there may be a moderate amelioration in the drop in vitamin D status compared to a control group who maintained their habitual lifestyle (*d* = 0.58, *p_x_* = 0.07). Furthermore, perhaps more importantly, the active vitamin D metabolite, 1,25(OH)_2_D_3_, was completely protected from a seasonal decline by exercise.

In the winter months, the amount of UVB radiation reaching the northern latitudes is insufficient for cutaneous synthesis of vitamin D.^[^ ^31^ ^]^ Accordingly, as anticipated, serum total 25(OH)D concentration decreased in both groups over 10 weeks of winter. While the control group displayed declines of 25% from baseline (14.0 ± 10.1 nmol L^−1^), the exercise group declined only 15% from baseline (8.3 ± 9.7 nmol L^−1^) a difference of 5.7 nmol L^−1^ [95% CI −0.2 to 11.5 nmol L^−1^]. Furthermore, regular exercise completely maintained circulating concentrations of 1,25(OH)_2_D_3_ during winter. The vitamin D receptor is present in almost all human cells, such that vitamin D plays a role across multiple organ systems,^[^ ^2^ ^]^ and it is principally 1,25(OH)_2_D_3_ that binds to the VDR to regulate gene transcription in target cells, with a binding affinity 1000× greater than 25(OH)D.^[^ ^2^, ^32^ ^]^ Vitamin D supplementation generally does not positively impact circulating 1,25(OH)_2_D,^[^ ^33^, ^34^ ^]^ unless supplementation is accompanied with high levels of physical activity, for example, in military recruits^[^ ^35^ ^]^ or in athletes.^[^ ^36^ ^]^ Furthermore, oral vitamin D_3_ supplementation also does not increase circulating 25(OH)D concentrations in people with overweight as effectively as in people with normal weight.^[^ ^37^ ^]^ Moreover, even when serum 25(OH)D status has been substantially improved with supplementation, nonclassical actions of vitamin D such as improvement muscle strength or reduced cancer incidence are not consistently demonstrated.^[^ ^38^, ^39^ ^]^ Thus, exercise has specific positive effects on vitamin D metabolism that is not achieved with vitamin D supplementation alone.

The maintenance of 1,25(OH)_2_D_3_ with exercise in overweight and obese people over winter is a novel finding. The half‐life of 1,25(OH)_2_D_3_ is only 4–6 h,^[^ ^40^ ^]^ so the present data likely represent chronic differences in 1,25(OH)_2_D_3_ between groups rather than any persistent acute exercise effect. Indeed, we previously demonstrated that a 60‐min bout of moderate intensity treadmill exercise increased the serum concentration of 1,25(OH)_2_D_3_ immediately and 1‐h‐post exercise, with concentrations returning to baseline within 24 h.^[^ ^15^ ^]^ In the present study, samples were taken 36 h after the last exercise bout. A logically appealing explanation for the maintenance of 1,25(OH)_2_D_3_ might be greater availability of the substrate (25(OH)D) owing to the moderate effect of exercise in maintaining vitamin D status. However, 1,25(OH)_2_D_3_ is considered to be tightly regulated and not positively associated to 25(OH)D concentrations.^[^ ^41^ ^]^ Indeed, the change in 1,25(OH)_2_D_3_ was not correlated with change in 25(OH)D in either group in the present study, replicating observations within the context of vitamin D_3_ supplementation.^[^ ^33^ ^]^ However, it is possible that the decrease in 25(OH)D availability increased renal production of 1,25(OH)_2_D contributing to its maintenance over winter.^[^ ^42^ ^]^ Importantly, circulating 1,25(OH)_2_D_3_ concentrations are also dependent on PTH and fibroblast growth factor 23 (FGF23), with both of these hormones themselves being regulated by 1,25(OH)_2_D as part of endocrine feedback regulation.^[^ ^20^ ^]^ While the present data do not show a persistent increase in circulating PTH, the accumulation of repeated acute responses of PTH to exercise suggest further investigation is needed to understand the dynamic relationship between these biomarkers.^[^ ^43^ ^]^ In future studies, it will also be interesting to assess potential effects of exercise on levels of FGF23 as a suppressor of 1,25(OH)_2_D production via inhibition of CYP27B1 expression. It should be noted that a previous study reported an increase in 1,25(OH)_2_D concentration following a weight loss with an exercise intervention, measured by immunochemiluminometric analyser.^[^ ^17^ ^]^ However, this study took place at latitude ≈37° N so the contribution of cutaneous synthesis cannot be ruled out,^[^ ^31^ ^]^ supplementation does not appear to have been controlled,^[^ ^44^, ^45^ ^]^ and 25(OH)D was not measured. Furthermore, the extreme range of lean mass changes reported, from losses over 10 kg to gains approaching 20 kg^[^ ^17^ ^]^ adds further complexity to interpretation of these data owing to the potential role of muscle in vitamin D storage.^[^ ^46^ ^]^ Irrespective of the mechanism, the biological implications for health from maintaining circulating 1,25(OH)_2_D_3_ concentration with exercise over the winter could be broad and wide reaching.^[^ ^47^ ^]^

It is well accepted that low vitamin D status is associated with greater absolute increases in serum 25(OH)D following supplementation.^[^ ^37^ ^]^ Here, we present the first evidence that *higher* vitamin D status (serum total 25(OH)D) is associated with greater absolute *declines* in serum 25(OH)D concentration over winter in the absence of supplementation. This may be due to first order kinetics but may also suggest that the rate of seasonal decline in vitamin D status is not linear. Serum 25(OH)D concentration may fall more rapidly at the beginning of winter, i.e., when vitamin D status is likely to be higher compared to later in winter. There may be common negative feedback mechanisms explaining these associations, underpinned by the priority to maintain calcium homeostasis,^[^ ^48^ ^]^ such that this is not simply regression to the mean.^[^ ^49^ ^]^ In any case, both groups were studied evenly across the winter and neither changed dietary vitamin D intake, so the decreases reflect the average rates of decline over the whole winter for each group. Extrapolating these rates predicts falls of 36.4 nmol L^−1^ in the control group and 21.6 nmol L^−1^ in the exercise group over the full winter. The control group would have ended the winter vitamin D deficient (20.6 nmol L^−1^), and the exercise group would have been insufficient (32.2 nmol L^−1^). Previous estimates from cross‐sectional data (not excluding people supplementing with vitamin D) suggest serum 25(OH)D concentration decreases by 37%–50% over winter in the UK.^[^ ^50^, ^51^ ^]^ The extrapolations from the present study suggest that decreases of 64% could be expected over winter without supplementation, compared to 40% if people undertook regular exercise aligned to the UK CMO and WHO guidelines.^[^ ^52^, ^53^ ^]^ These predictions should be interpreted cautiously given the inherent assumptions, but it provides a useful indication of the potential magnitude of the effect.

Other vitamin D metabolite concentrations declined over winter in the absence of supplementation (specifically 3‐Epi‐25(OH)D_3_, vitamin D_3_, free 25(OH)D, and bioavailable 25(OH)D), while 25(OH)D_2_ remained stable. There was no effect of the exercise intervention on changes in the concentration of these metabolites and markers. However, exercise over winter had divergent effects on correlations between concentrations and changes in concentrations in vitamin D metabolites. For example, there was a strong negative correlation between serum 25(OH)D concentration and change in 25(OH)D in the control group that did not exist in the exercise group. Declines in serum 25(OH)D were also strongly correlated with 24,25(OH)_2_D_3_, 3‐Epi‐25(OH)D, serum vitamin D_3_, and adipose vitamin 25(OH)D_3_ in the control group but not the exercise group. Conversely, there were strong negative correlations between change in serum vitamin D_3_, and serum concentrations of 25(OH)D, 24,25(OH)_2_D_3_, 3‐Epi‐25(OH)D, and vitamin D_3_, and adipose vitamin D_3_ concentrations in the exercise group that were not present in the control group (Figure 5). This supports the notion that exercise may independently affect the negative feedback loops controlling circulating 25(OH)D and vitamin D_3_ concentrations. It also highlights that different vitamin D storage compartments, and indeed metabolites within those compartments, may respond differently to exercise.

When high systemic 25(OH)D concentrations are present, 24‐hydroxylase activity upregulation increases conversion of 25(OH)D into 24,25(OH)_2_D_3_, and during hypovitaminosis 24‐hydroxylase is downregulated, preserving 25(OH)D.^[^ ^20^ ^]^ In the present study, while there was a moderate effect for the maintenance of 24,25(OH)_2_D_3_ with exercise, this was not statistically significant. However, the relative changes in 25(OH)D, 1,25(OH)_2_D_3_, and 24,25(OH)_2_D_3_ resulted in significant time effects on vitamin D metabolite ratios (VMR). The 1,25(OH)_2_D_3_:24,25(OH)_2_D_3_ VMR increased in both groups over winter, suggesting preferential conversion of 25(OH)D to 1,25(OH)_2_D_3_, which is expected with declining serum 25(OH)D concentration.^[^ ^42^ ^]^ However, there was no suggestion that exercise altered this VMR despite the maintenance of 1,25(OH)_2_D_3_. Importantly, this significant increase in the 1,25(OH)_2_D_3_:24,25(OH)_2_D_3_ VMR suggests that an overcompensation of 24‐hydoxylase activity previously reported with elevated serum 1,25(OH)_2_D concentrations following a high dose supplementation in athletes did not occur with exercise in the present study.^[^ ^36^ ^]^ This may suggest that exercise in winter supports more “efficient” vitamin D metabolism, making better use of the available substrate to generate the active metabolite without tipping the balance into a negative feedback loop associated with increased systemic 25(OH)D concentration.

With the preservation of serum 1,25(OH)_2_D_3_ concentration with exercise over winter, and an ameliorated decline in 25(OH)D, it might be expected that the rate of 25(OH)D expenditure would have changed too. However, the half‐life of the ingested d_3_‐25(OH)D_3_ tracer did not change over winter, or in response to exercise. Half‐life was ≈14 d, as has been seen previously in both UK and Gambian populations,^[^ ^21^, ^54^ ^]^ and it seems that the rate of 25(OH)D expenditure is preserved even during periods of high energy expenditure. This is coherent with evidence that neither pregnancy nor breastfeeding appear to change circulating 25(OH)D_3_ half‐life either.^[^ ^55^ ^]^ However, in the context of a 14‐day half‐life of 25(OH)D in serum, the absolute declines in 25(OH)D status observed in the present study support that mobilization of endogenous vitamin D stored in tissues must play a role in preventing even more substantial declines in vitamin D status,^[^ ^50^ ^]^ particularly given the low dietary vitamin D intake reported in both groups.

A primary site of vitamin D accumulation is adipose tissue.^[^ ^7^, ^8^, ^56^, ^57^ ^]^ Adipose tissue concentrations of 25(OH)D_3_ declined by 16 ± 19% and 23 ± 17%, with adipose vitamin D_3_ concentration declining by 15 ± 38% and 10 ± 22% in the control and exercise groups, respectively, with no statistical difference between groups. Evidence from supplementation studies in vivo confirm that adipose tissue sequesters excess vitamin D, with vitamin D_3_ concentrations generally reported to be 10× greater than 25(OH)D concentrations.^[^ ^7^, ^57^ ^]^ Adipose tissue vitamin D concentrations decrease when high serum levels are not maintained,^[^ ^58^ ^]^ either by release into the circulation^[^ ^56^ ^]^ or potentially by local conversion and use.^[^ ^59^ ^]^ The present study is the first direct measure of adipose 25(OH)D and vitamin D_3_ concentrations decreasing over the winter. In vitro evidence supports the concept that, even in people with overweight, vitamin D can be mobilized from adipose tissue through lipolytic stimuli such as exercise.^[^ ^14^ ^]^ As such, we speculated that maintenance of serum vitamin D status would be via the mechanism of vitamin D mobilization from adipose. It is therefore interesting that exercise did not drive a greater decrease in adipose tissue concentrations of vitamin D. There was no correlation between the change in serum 25(OH)D and changes in adipose vitamin D_3_ concentrations in either group. So, while it is clear that vitamin D sequestered in adipose is released over winter, it is not clear precisely how or if this contributes to the maintenance of vitamin D status. Future research should employ additional tracer approaches to examine tissue specific vitamin D metabolism and identify relative contributions of different storage compartments to the maintenance of serum 25(OH)D and 1,25(OH)_2_D concentration.

Weight loss has an inverse relationship with improved vitamin D status.^[^ ^9^ ^]^ In the VitaDEx study, body mass was deliberately maintained during the exercise intervention through increased energy intake to isolate the effect of exercise on vitamin status. Given that total fat mass remained stable, lipid droplets within adipocytes were presumably replenished and so perhaps the mechanism by which circulating vitamin D accumulates in adipocytes was simultaneously activated. It may be that exercise does release vitamin D from adipose, but subsequent reuptake cancels out longitudinal changes, and indeed minor fluctuations in adipose mass itself might play a role in this mechanism. Certainly, it appears that 25(OH)D and 1,25(OH)_2_D_3_ do not remain elevated in the circulation for long after a single bout of exercise.^[^ ^15^ ^]^ Decreases in intrahepatic fat content have been associated with increased serum 25(OH)D concentration in elderly men following a five‐week exercise intervention.^[^ ^16^ ^]^ Omental adipose tissue is particularly responsive to adrenergic activation^[^ ^12^ ^]^ and the current study only assessed 25(OH)D and vitamin D_3_ in subcutaneous stores. Thus, a key adipose compartment may have been missed. It should also be highlighted that, if vitamin D metabolism occurs within adipose tissue,^[^ ^59^ ^]^ as the observed adipose expression of *CYP27B1* and *CYP2R1* suggests, then making inferences regarding vitamin D release from adipose based purely on metabolite concentration may be an oversimplification. For example, a *p‐*value of 0.052 for an interaction effect in *CYP2R1* expression in adipose between groups over the intervention period was observed. If this led to a greater local 25‐hydroxylase activity in the control group, then increased vitamin D3 conversion to 25(OH)D within adipose tissue could be expected. The fate of locally produced 25(OH)D is unclear, but this could mean that changes in adipose tissue concentrations of each metabolite may therefore reflect the combination of release of metabolites, and their local conversion to other metabolites.

There are a number of limitations with this study to acknowledge. Two of the three winters of data collection took place in the context of COVID‐19 restrictions. Despite successfully implementing strategies to ensure the study could continue within the ever‐changing guidelines, the study was undoubtedly affected. The original protocol included muscle biopsy samples, however, due to logistical constraints owing to COVID‐19 restrictions, this measurement had to be removed. Muscle is a dynamic storage site of vitamin D, so these data may have been particularly informative in identifying vitamin D flux between storage compartments.^[^ ^46^ ^]^ Moreover, the principal organs involved in vitamin D metabolism are the liver and kidney.^[^ ^41^ ^]^ While systemic AST and ALT activities in each group suggest that liver function was healthy, and not different between groups or over the intervention period, these markers do not provide insight into altered vitamin D metabolism at these sites. However, this study also has several methodological strengths that warrant highlighting. The RCT design and rigorous control of extraneous variables, such as body mass and cutaneous vitamin D synthesis, provide confidence in the inferences around in vivo biological mechanisms of exercise in a free‐living setting. Moreover, the use of LC‐MS/MS to measure vitamin D metabolites beyond 25(OH)D in both serum and adipose, provides novel insight into to the complexities and dynamic nature of vitamin D metabolism over winter and in the context of exercise. Similarly, measurement of gene expression in adipose tissue, and systemic vitamin D expenditure rates, provides clues as to physiological processes that may, or may not, be altered by exercise without weight loss during the winter. This is supported by the range of measures in place to ensure intervention adherence, both to the undertaking of the exercise and the maintenance of body mass, and the effectiveness of the intervention at increasing physical activity levels and improving maximal rate of fat oxidation and V̇O_2_max. The weekly total duration of programmed exercise was 166 ± 16 min, aligning with the WHO physical activity guidelines.^[^ ^53^ ^]^ This broadly achievable increase in habitual physical activity further supports the translation of these findings to real world. Understanding the downstream impact that maintained circulating 1,25(OH)_2_D_3_ concentration or vitamin D mobilized from adipose tissue by exercise, weight loss, or both, has on health markers such as metabolic and immune function or bone health, compared to vitamin D supplementation, is an important area for further research.

In conclusion, moderate intensity exercise without weight loss maintains the serum concentration of the active vitamin D metabolite 1,25(OH)_2_D_3_ in nonsupplementing individuals with overweight and obesity compared to a decline in the control group over 10 weeks of winter. Exercise also moderately ameliorated the seasonal decline in vitamin D status measured as serum 25(OH)D despite not altering the decline in adipose vitamin D metabolite concentrations, expression of genes in adipose related to vitamin D metabolism, or half‐life of 25(OH)D in plasma. Supplementation strategies are known to be less effective in people with overweight and obesity, and supplementation does not improve 1,25(OH)_2_D concentration. Thus, the findings of the VitaDEx RCT point to novel mechanisms by which exercise could have important public health implications via positive effects on vitamin D metabolism.

## 4 Experimental Section

### Experimental Model and Study Participant Details—Human Subjects

Participants were recruited through public advertisement via the University of Bath homepage, through social media, and through direct mailing by local primary care practices to potentially eligible research participants based on patient database screening by research nurses. Recruitment commenced in April 2019. Expressions of interest in participation (*n* = 1033) were received by email and phone to Dr. Oliver Perkin and Miss Sophie Davies. Subsequently, 120 potential participants underwent screening. Inclusion criteria were: aged 25–65 years of age, non‐smoking for at least 6 months prior to enrolment, fat mass index (fat mass kg/height in m^2^; FMI) of >7.5 kg m^−2^ (♂) and >11 kg m^−2^ (♀), self‐report engaging in no vigorous activity and less than 150 minutes of moderate intensity activity in an average week, and an objectively measured physical activity level (total energy expenditure/resting energy expenditure (PAL)) of less than 2.00. Exclusion criteria were: consumption of dietary supplements containing vitamin D or use of sunbeds within three months starting and during the study; use of weight loss drugs, >5% change in body mass, or large change in habitual lifestyle in previous 6 months; diagnosed coronary heart disease, chronic kidney disease, type II diabetes, stroke, heart failure, peripheral arterial disease, “severe hypertension” (blood pressure greater than 180/110 mmHg at rest), or positive responses to the Physical Activity Readiness Questionnaire (PAR‐Q);^[^ ^60^ ^]^ participation in another interventional research trial or lifestyle supportive intervention within two months of enrolment; use of medication that might interfere with the study outcomes based on evidence available in the British National Formulary (BNF)^[^ ^61^ ^]^ at commencement of the study data collection; sensitivity or allergy to lidocaine or any local anesthetic medicines; pregnancy; or inability to change physical activity levels. Participants refrained from donating blood while participating in the study. A total of 51 participants were included and randomized to one of two study groups: a lifestyle maintenance (control) group (*n* = 21), or an exercise group (*n* = 30). All participants provided written informed consent prior to participation in the VitaDEx study. The VitaDEx study was approved by the Wales NHS Research Ethics Committee 5 Bangor (18/WA/0392). Data collection took place between September 2019 and April 2022.

### Experimental Model and Study Participant Details—Experimental Design

The study was a single‐center, randomized controlled trial with two groups undertaken in the Department for Health, University of Bath, UK, in accordance with the Helsinki Declaration. The exercise intervention/control period was 10 weeks during the winter, with collection of primary and secondary outcome data was scheduled to take place from October to March inclusive. Baseline assessment was scheduled to achieve an equal spread of participants from each group in each month of winter. Screening could take place all year round, however self‐report lifestyle behaviors were re‐checked at baseline if screening had taken place in the summer month.

The study involved two screening assessments with a week of habitual physical activity monitoring prior to randomization. The first screening session involved completion of self‐report eligibility questionnaire and equipping potential participants with a physical activity monitor, and the second involved assessment of resting metabolic rate, basic anthropometry and body composition, blood pressure, and individual calibration the physical activity monitor. Study group allocation by minimization was based on data collected during screening visits, and once allocation had occurred, the participant counted toward the total sample size. Participants and researchers were informed of their allocation.

**Figure 6:**
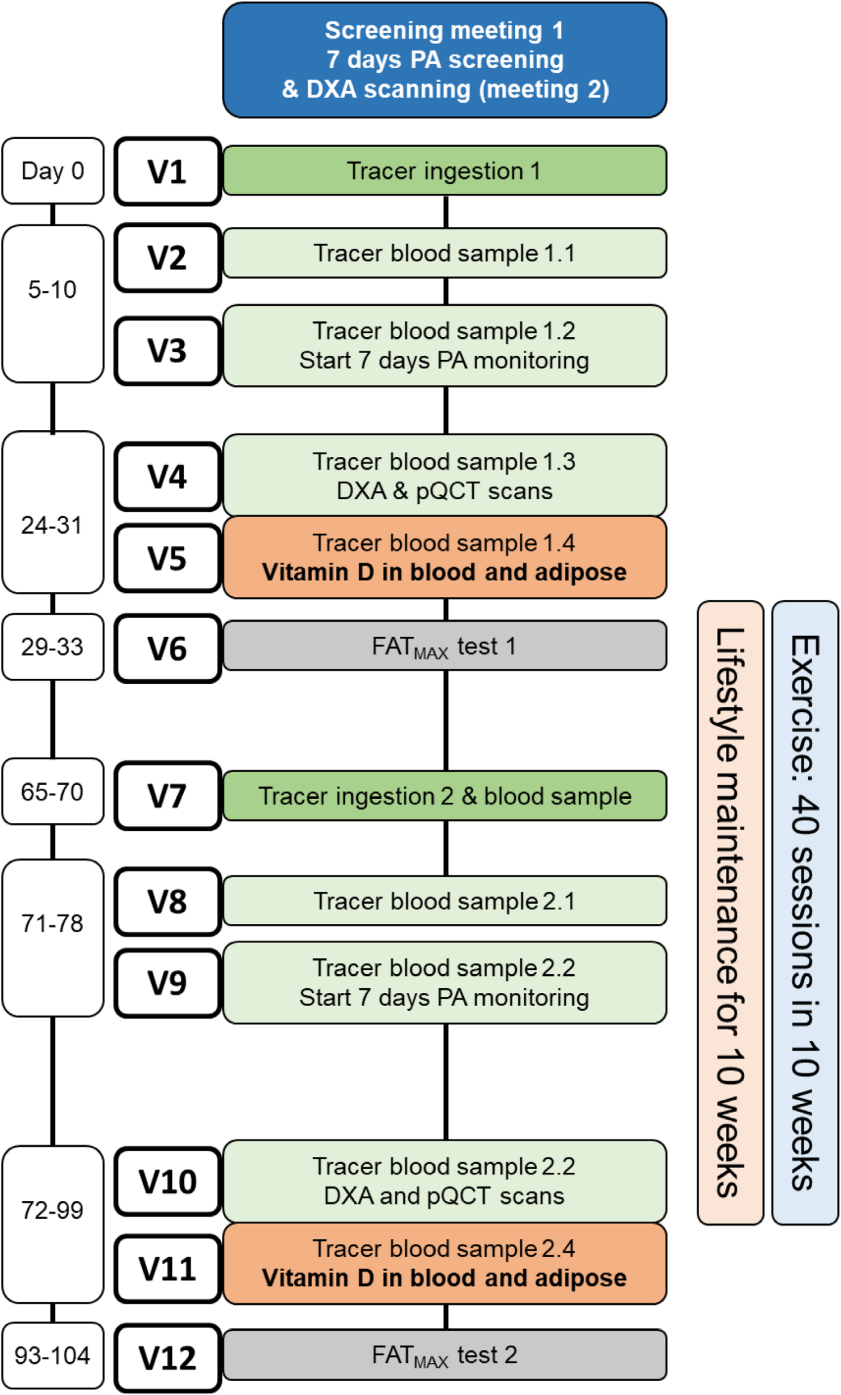
A schematic overview of the study design for participants completing the tracer assessment of whole‐body vitamin D expenditure rate. For participants not undertaking this measurement, the trial was completed in six or seven laboratory visits, matching the same timeline for measurement and intervention periods. DXA, dual‐energy X‐ray absorptiometry; PA, physical activity monitoring with Actiheart 5; pQCT, peripheral quantitative computed tomography; FAT_MAX_, treadmill‐based incremental exercise test.

All participants undertaking the tracer assessment of whole‐body vitamin D expenditure rate (*n* = 24; control group *n* = 10, exercise group *n* = 14) attended the laboratory on 12 occasions over ≈14 weeks (see **Figure** **6**). During the first trial visit, participants consumed a breakfast meal containing a stable isotope vitamin D tracer, demarcating the beginning of the trial for that participant. All participants maintained their habitual lifestyle for approximately four weeks after tracer ingestion, with two laboratory visits for 4‐h fasted blood samples scheduled five to ten days after ingestion to assess tracer incorporation into plasma. Baseline physical activity for 7 d and dietary intake over 3 d were measured after these laboratory visit. The next two visits were scheduled 24–31 d after tracer ingestion. On the fourth visit, following an overnight fast and void, body composition was assessed and another blood sample for plasma trace concentration was obtained. On the fifth visit, following an overnight fast, a blood sample for serum concentrations of 25(OH)D and secondary vitamin D metabolites, and plasma tracer concentrations, and other confirmatory blood markers was collected, along with an adipose tissue biopsy. This visit marked the beginning of the 10‐week intervention phases. One‐to‐three days later, participants attended the laboratory for assessment of baseline cardiovascular fitness on a treadmill. This visit was considered the first exercise training session for participants in the exercise group, with a further exercise test on an exercise bike scheduled one‐to‐three days after which was considered the second exercise session. The individualized exercise program was generated from the data collected at these sessions, with participants continuing the exercise intervention aiming for four exercise sessions per week. Approximately 6 weeks later, these six visits were repeated with the same intervals as described above, starting with a second tracer ingestion, such that the second main trial day fell 10 weeks after the first. For participants in the exercise group, at the eighth and ninth visit, exercise tests were repeated to adjust the exercise program accordingly, with both tests counting as exercise sessions. For participants not undertaking tracer assessment of whole‐body vitamin D expenditure rate, the trial was completed in six to seven visits.

For participants not undertaking the vitamin D expenditure element of the study laboratory visits that were principally for collection of blood samples for plasma tracer concentration measurement could be omitted. As such, baseline data were collected in three laboratory visits across 3–6 d; one visit for anthropometry, one for blood and adipose tissue sampling, and one for cardiovascular fitness assessment. Three additional laboratory visits were scheduled for a baseline exercise bike fitness test, and for mid‐intervention treadmill and exercise bike fitness tests. Post‐intervention assessments were taken across three visits as described for baseline assessment, such that the blood and adipose tissue sampling took place 10 weeks after the baseline samples were collected.

### Experimental Model and Study Participant Details—Protocol Changes due to the COVID‐19 Outbreak

Laboratories were closed on March 12, 2020 due to COVID‐19 lockdown restrictions, forcing the withdrawal of three participants part way through the intervention who could not complete post‐intervention assessment (see Figure 1). As such, a protocol amendment to extend the sample size to 53 participants was approved by the ethics committee. Allocation of participants to study groups was on a 1:1 basis in the first winter of testing (October 2019 to March 2020), however with COVID‐19 lockdown restrictions in place for the second and third winters disproportionately impacting feasibility of participation in the exercise group, allocation was amended to 1:2 (control:exercise). This ensured adequate sample size was achieved in both groups. As described, to complete the vitamin D tracer expenditure measurement, 12 visits to laboratory were required, whereas the primary, secondary, and confirmatory outcome data could be collected in six visits, plus three additional visits for exercise group participants. As such, with limitations of personnel numbers in laboratories to adhere to social distancing measures, it was not feasible to collect these data from all participants. Furthermore, to accommodate participation in the study for participants not comfortable or able to access gyms for exercise, laboratory exercise equipment was loaned out to participants to exercise in their homes.

### Method Details—Physical Activity Monitoring

Habitual physical activity was assessed over seven full 24‐h days of free living at screening, and prior to baseline and post‐intervention blood sample collection, using a chest‐mounted accelerometer with integrated heart rate recording (Actiheart 5; CamNtech, Cambridge, UK). The device was worn continuously apart from during water‐based activities such as bathing or swimming, with movement and heart rate data collected in 15‐s epochs. Participants were instructed to continue with their current lifestyle while wearing the device, and to wear the device for exercise sessions if the wear period was during the intervention for exercise group participants.

The Actiheart 5 was individually calibrated using resting metabolic rate (RMR) and heart rate data from treadmill exercise tests. For screening assessment, resting metabolic rate was measured for 20 min using the GEM indirect calorimeter (GEM Nutrition Ltd., Cheshire, UK). Measurements were taken with the participant in supine rest in a quiet room, in the fasted state, after 10 min of rest and habituation to the measurement hood, and before any other measures that could influence metabolism. For the other two wear periods, RMR was estimated from the Schofield equation factoring sex, age, and body mass.^[^ ^62^ ^]^ Heart rate and energy expenditure during physical activity to individually calibrate the Actiheart 5 were collected during incremental exercise tests. For each wear period, a new calibration was conducted using data from the temporally closest treadmill test, i.e., the baseline and post‐intervention cardiovascular fitness test data was used to calibrate the device for the baseline and intervention physical activity monitoring periods. A separate treadmill walk test was conducted for calibration for the screening physical activity monitoring period. This involved four stages of four minutes of treadmill walking at self‐selected intensities eliciting ≈10 beats min^−1^ difference in heart rate between stages. During the final minute of each stage, expired air was collected using the Douglas bag method to estimate energy expenditure via indirect calorimetry. Expired gas was analyzed using a Servomex 1400 gas analyser (Servomex Ltd., UK) adjusting for atmospheric CO_2_.^[^ ^63^ ^]^

### Method Details—Dietary Assessment of Vitamin D Intake

During the week of physical activity monitoring before baseline and post‐intervention measures, participants completed three‐day weighed food and fluid intake diaries, recording two weekdays and one weekend day. Food diaries were analyzed using a commercially available online platform (Nutritics Research Edition v5.099, Nutritics Education, Ireland) and daily vitamin D intake data were extracted. The food diary data was also used to inform individualized recommendations for food choices which could replace extra energy expended during exercise sessions for participants in the exercise group.

### Method Details—Anthropometry Assessment

Body mass was recorded using digital weighing scales (BC543, Tanita, Amsterdam, Netherlands) with the participant having voided and removing shoes and heavy clothing. Height was recorded with a stadiometer (222 Seca, Hamburg, Germany) while the participant standing in the Frankfort plane without shoes. Body mass recorded at every lab visit for all participants to ensure weight maintenance across the intervention period.

### Method Details—Body Composition Assessment

Body composition was assessed using DXA (Discovery; Hologic, Bedford, UK) during screening, and baseline and post‐intervention. Participants arrived for scanning following an overnight fast, having drunk a pint of water between waking and arrival, and voided before the scan. Participants removed all metal items and wore loose fitting clothes, with clothing replicated on repeated scanning occasions. Whole body DXA scans were undertaken while the participant remained motionless, laying supine on the scanning bed. Manual placement of boundaries between discrete anatomical regions was conducted for all scans by the same investigator (OJP) before analysis using manufacturer's software. Shank (tibia) bone and muscle characteristics (density and geometry) of the right leg were measured using peripheral quantitative computed tomography (pQCT, XCT3000, StraTec Medizintechnik GmbH, Pforzheim, Germany) at baseline and post‐intervention. Scans were conducted with participants lying supine on a physiotherapy bed, bare from the knee down. A single slice scan at the 38% distal limb length was used to analyze cortical bone mineral density (BMD), and shank muscle cross sectional area (mCSA) was analyzed from a single slice scan at 66% distal limb length. Scans were analyzed using the BoneJ plugin (Version 1.4.2) for ImageJ (1.51j8, Wayne Rasband, National Institutes of Health, USA).^[^ ^64^, ^65^ ^]^

### Method Details—Blood Samples

Venous blood was collected by venepuncture from an antecubital vein. Blood samples for biochemical markers analyzed on plasma were collected into EDTA coated tubes (Sarstedt, Germany), and immediately centrifuged for 10 min at 4000 *g* in 4 °C. Blood samples for analysis of serum marker concentrations were collected into uncoated tubes (Sarstedt, Germany) and left to clot at room temperature for 30 min before centrifugation for 10 min at 4000 *g* in 4 °C. Blood samples for the measurement of d3 25(OH)D_3_ tracer enrichment in plasma were collected in lithium heparin tubes (Becton Dickinson Vacutainer, UK) and immediately centrifuged for 10 min at 4000 *g* in 4 °C. Aliquots of supernatant were immediately frozen on dry ice before storing at −70 °C until analysis.

### Method Details—Stable Isotope Tracer

Vitamin D half‐life was measured by giving participants a low oral dose of a stable isotope of vitamin D “tracer” d_3_‐25(OH)D_3_; 25(OH)D_3_ labeled in nonmetabolically active positions (deuterated (6, 19, 19) 25‐hydroxy vitamin D3 (product number: 705888; 97 at%; purity 98%; Sigma‐Aldrich, Poole, UK)).^[^ ^54^, ^55^ ^]^ The size of the tracer dose was selected to result in plasma concentrations just above the lower limit of detection of the analytical method to minimally perturb serum 25(OH)D concentration. The dose was 60 nmol (24 µg), equivalent to the dose in many daily vitamin D supplements. The tracer was dissolved in 1 mL olive oil, poured onto on a small piece of ciabatta bread, and given with a standard breakfast with a fat content of 20 g to ensure reliable absorption of the tracer.^[^ ^54^, ^55^ ^]^ Participants were asked not to eat or drink anything (except water), and refrain from lying down and strenuous exercise for 3 h following the dose.

Four blood samples were collected to detect plasma d_3_‐25(OH)D_3_ tracer content over the 31 d following tracer ingestion, with samples collected after a minimum 3‐h fast. With a degree of flexibility around the scheduling relative to the tracer administration, two blood samples were collected 5–10 d after ingestion, and two collected 24–31 d after ingestion. An additional blood sample was taken at the second tracer ingestion (approximately intervention week six) to assess any residual tracer in blood plasma from the first tracer‐testing period.

### Method Details—Adipose Tissue Biopsy

Subcutaneous abdominal adipose tissue was biopsied 4–7 cm lateral of the umbilicus. The area was thoroughly disinfected with Videne, before injection of anesthetic (≈3 mL lidocaine hydrochloride 1%) into a small area. Five minutes later, a 14‐gauge needle was inserted into the subcutaneous adipose tissue around the waist, and ≈1–2 g of adipose tissue collected using the needle aspiration technique.^[^ ^66^ ^]^ Adipose tissue biopsies were cleaned of blood using saline (0.9% NaCl) separated into aliquots and immediately frozen on dry ice before storing at −70 °C until analysis.

### Method Details—Cardiovascular Fitness Testing

At baseline and post‐intervention, all participants completed a maximal walking exercise test on a motorized treadmill following an overnight fast, having consumed a pint of water between waking and arrival to ensure hydration status. The protocol was adapted from Achten et al.^[^ ^67^ ^]^ with longer stages for individuals with lower cardiorespiratory fitness.^[^ ^68^ ^]^ Walking began at 3.5 km h^−1^ with the gradient set at 0% incline, for 4 min. After this point, the treadmill speed was increased to 4.5 km h^−1^, with gradient remaining at 0% incline, for 4 min. Thereafter, treadmill speed remained constant at 4.5 km h^−1^ for the rest of the test, while gradient increased by 4% incline every 4 min for a further three stages, until volitional exhaustion was reached or 20 min of walking had been completed, in which case the treadmill gradient was increased by 2% min^−1^ until volitional exhaustion. Expired gas samples were collected using a Douglas bag in the final minute of the first five stages, and the final 30 s of each 1‐min stage completed thereafter, until volitional exhaustion. Maximal rate of fat oxidation (MFO) and maximal rate of oxygen consumption (V̇O_2_max) were estimated using indirect calorimetry based on the assumptions from Frayn (1983).^[^ ^69^ ^]^ Expired gas was analyzed using a Servomex 1400 gas analyser (Servomex Ltd., UK) adjusting for atmospheric CO_2_.^[^ ^63^ ^]^ Heart rate (HR) via telemetry (RS400; Polar, Kempele, Finland) and ratings of perceived exertion (RPE) on the Borg scale^[^ ^70^ ^]^ were recorded during each expired gas sample. Verbal encouragement was provided throughout the test, and participants were allowed ad‐libitum water intake and use of fans for cooling. Attainment of a valid V̇O_2_max was assumed if at least one of the four following criteria were met in the final stage of exercise: i) reaching age‐predicted maximal heart rate (±10 bpm), ii) measured respiratory exchange ratio (RER) >1, iii) RPE of 20 reported, or iv) V̇O2 plateau (<150 mL change per min) compared to the penultimate stage.

Participants in the exercise group also completed cardiovascular fitness testing on an exercise bike in week 1 and week 6 of the intervention. The exercise bike ramp test started at a resistance of 30 W and increased by 1 W every 3 s until volitional exhaustion. Indirect calorimetry using a breath‐by‐breath gas analysis system (TrueOne 2400; Parvo Medics, Salt Lake City, UT) was used to estimate V̇O_2_max, and real‐time HR was recorded via telemetry (RS400; Polar, Kempele, Finland) in the Parvo Medics software.

### Method Details—Exercise Intervention

Participants in the exercise group undertook 10 weeks of multimodal cardiovascular exercise training approximately four times per week, with the aim to complete 40 exercise sessions. The last exercise session took place >36 h before the post‐intervention measures. The four cardiovascular exercise tests described previously counted toward the 40 sessions. Participants received a three‐month gym membership so that all exercise sessions took place indoors to avoid sunlight exposure. Some participants exercised in their own homes with loaned exercise equipment. The four weekly sessions included two treadmill walking sessions, one steady state exercise bike session, and one low‐volume high intensity interval training (LV‐HIIT) session on an exercise bike. The four sessions could be undertaken in any order, with all four sessions completed before restarting the four‐session cycle, with exercise taking place on no more than two consecutive days to maintain regular training frequency. The exercise durations and intensities varied between training sessions and were personalized to each participant and progressive across the intervention. The overall aim of the exercise intervention was to increase energy expenditure with a specific focus on lipid metabolism during exercise, and secondarily to improve cardiovascular fitness (MFO and V̇O_2_max). The variety of exercises and intensities were intended to reduce boredom and monotony, and in particular, the range in session duration was intended to allow flexibility in scheduling to facilitate adherence. The treadmill sessions were undertaken at an exercise intensity corresponding to MFO (Fat_max_) based on HR. The duration of each session was set to achieve a pre‐determined energy expenditure relative to body mass in each session (12 kJ kg^−1^ progressing to 15 kJ kg^−1^). The exercise bike session was undertaken at intensities based on V̇O_2_max based on HR (60% progressing to 75% V̇O_2_max) for set durations (30 min progressing to 40 min). The LV‐HIIT session consisted of repeated “sprints” (eight progressing to 10) of 1 min of cycling at 80–100 rpm at a resistance to illicit 90%–95% of maximum heart rate (HR_max_), followed by 1 min of light cycling.^[^ ^71^ ^]^ Participants wore a HR monitor (TickrX; Wahoo Fitness, Atlanta, Georgia, USA) with a wrist worn watch to display HR during exercise (Forerunner 25; Garmin, Olathe, Kansas, USA). Heart rate was used to guide training session intensity for pragmatism and as it would inherently induce progression in absolute training intensity as exercise capacity increased with training.

Four concurrent strategies were used to monitor intervention adherence; i) in person or video calling supervision was offered for all sessions, ii) participants completed a weekly training logbook which set out the training intensities and durations by week, iii) the TickrX monitor logged the occurrence of exercise sessions, and iv) the lead researcher contacted all participants by email weekly to ask how many exercise sessions had been completed that week. Weekly exercise energy expenditure was estimated based on the cardiovascular fitness test data and individualized exercise programming. To ensure weight maintenance in the exercise group across the intervention, options of specific quantities of foods habitually consumed based on an individual participant's food diary were recommended on a daily basis. This approach was taken to avoid introducing any systematic bias associated with supplying the intervention group supplemental food to replace the extra energy expended through exercise. In either group, if weight loss >2% of body mass was recorded between laboratory visits then participants would be recommended to increase their energy intake.

### Method Details—Biochemical Analysis of Vitamin D Metabolites in Serum and Adipose Tissue

The analysis of vitamin D metabolites was undertaken by researchers blinded to experimental conditions.

Serum vitamin D metabolites (25(OH)D_2_, 25(OH)D_3_, 1,25(OH)_2_D_3_, 24,25(OH)_2_D_3_ and 3‐epi‐25(OH)D_3_) were measured with LC‐MS/MS^[^ ^19^ ^]^ at the ANZAC Research Institute, Concord NSW, Australia. 20 µL of internal standard (25(OH)D_3_‐13C5 (30 ng mL^−1^), 25(OH)D_2_‐d_3_ (5 ng mL^−1^), 3‐epi‐25(OH)D_3_‐d_3_ (8 ng mL^−1^), 24,25(OH)_2_D_3_‐d_3_ (8 ng mL^−1^), and 1,25(OH)_2_D_3_‐d_3_ (100 pg mL^−1^))) was combined with 300 µL of serum sample in 1.5 mL microcentrifuge tubes for vitamin D metabolite extraction. Samples underwent protein precipitation via the addition of 450 µL isopropanol/water (50/50 v/v), and vortexed for 10 min at high speed, then left for a further 15 min and centrifuged at 9000 rpm for 5 min. Supernatant was transferred to glass tubes for liquid–liquid extraction^[^ ^72^ ^]^ with some modifications. Extraction was carried out with the addition of 1 mL hexane vortexed for 30 s followed by the addition of 1 mL MTBE vortexed for 30 s, and samples were frozen at −20 °C for 2 h. The organic layer was transferred and evaporated to dryness under nitrogen at 50 °C. The dry residue samples were derivatized by adding 0.125 mg mL^−1^ PTAD dissolved in acetonitrile and incubating for 2 h in darkness at room temperature. The reaction was quenched with the addition of 20 µL water, the samples were then dried under nitrogen and reconstituted in 75 µL water/methanol (50/50 v/v) and transferred to a 96‐well microtiter plate. Analysis was performed on an SCIEX Exion LC system coupled to a mass spectrometer (SCIEX 6500 QTRAP), using electrospray ionization in positive mode. Multiple reaction monitoring mode was obtained with settings optimized for the various transitions by infusing pure standard for each analyte into the mass spectrometer. Unit mass resolution was used in both mass‐resolving quadruples Q1 and Q3, and a single qualifier and another quantifier ion (QI) were optimized for each analyte. During the sample run, the acquisition method was split into three periods to quantitate groups of metabolites based on retention time: period 1 was 0–8.6 min, period 2 was 8.6–16 min, and period 3 was 16–26.2 min. For liquid chromatography separation of metabolites, a Waters UPLC BEH Phenyl (2.1 × 75 mm, 1.7 µm) column was used, with column temperature set to 40 °C and flow rate of 0.300 mL min^−1^ with a mobile phase consisting of A:water 0.1% formic acid, B:methanol 0.1% formic acid with the following mobile phase gradient; 0 min: 38%‐A:62%B, 0.01–12 min: 35%‐A:65%B, 12.01–22.4 min: 28%‐A:72%B, 22.41–25 min: 28%‐A:72%B, 25.01–26.5: 38%‐A:62%B. Two gradient steps were used at 12.01–22.4 and 22.41–25 min to achieve 72% methanol mobile phase composition by 22.4 min and maintain this until 25 min into the sample run. The overall run time was 26.5 min. A 35 µL injection volume was used and the autosampler temperature set to 10 °C.

Serum samples at each timepoint were analyzed for vitamin D_3_ and d_3_‐25(OH)D_3_ (vitamin D tracer), and adipose tissue were analyzed for 25(OH)D_3_ and vitamin D_3_ by UPLC‐MS/MS^[^ ^73^ ^]^ at the Nutritional Biomarker Laboratory, MRC Epidemiology Unit, University of Cambridge, UK, based on a method described by Assar et al.^[^ ^74^ ^]^

To precipitate proteins from serum samples, 200 µL sample, 100 µL internal standard, and 200 µL 73% methanol (aq) were mixed, followed by liquid–liquid extraction using 1.5 mL hexane. After removal and drying of the solvent layer, 4‐phenyl‐1,2,4‐triazoline‐3,5‐dione (PTAD) derivatization was carried out using 50 µL 0.5 mg mL^−1^ in acetonitrile. 100 µL ethanol was used to quench the reaction. The samples were then dried and reconstituted in 100 µL 80% acetonitrile (aq), and transferred to HPLC vials, with 15 µL injected for analysis.

Saponification of adipose was performed using methods described by Best et al.^[^ ^7^ ^]^ Briefly, 10–15 mg of tissue was added to 200 µL sodium chloride (9 g L^−1^) and 200 µL 1 m sodium hydroxide and incubated for 60 min at 95 °C. 200 µL of internal standard was added to the solution before liquid:liquid extraction using 1 mL heptane/MTBE (1:1). Samples were snap frozen in a dry ice/methanol bath, decanted, and the solvent layer dried. Samples were reconstituted in 250 µL acetonitrile were decanted into clean glass tubes and dried before derivatizing with 50 µL PTAD (0.5 mg mL^−1^ in acetonitrile). 75 µL water was added, and the mixed solution transferred to HPLC vials for analysis using 37.5 µL injection volume.

For both serum and adipose tissue samples, detection of the analytes and internal standards were accomplished by LC‐MS/MS analysis using a Waters ACQUITY UPLC system coupled to an AB Sciex QTrap 5500 mass spectrometer. Ratio of analyte to internal standard signal was compared a calibration curve to determine analyte concentration. Analytes were resolved using reversed phase UPLC on a Waters Cortecs C18+ column 2.2 × 150 mm 1.6 µm column at 45 °C with a 17 min run time (0–5 min 80% A, 5.1 min 90% A, 10 min 94% A, 10.1–12 min 100% A to wash out column 12.1–17 min 80% A return to starting conditions). Mobile phase A used methanol with 0.1% formic acid, and Mobile phase B used water with 0.1% formic acid.

### Method Details—Confirmatory Biochemical Measures

All rested and overnight fasted samples were run in duplicate, and to avoid inter‐assay variation, each participant's baseline and post‐intervention samples were analyzed in the same run. Serum concentration of DBP was analyzed using a commercially available ELISA (Immundiagnostik, Germany). Intact plasma PTH was analyzed using a Roche Cobas analyser (electrochemiluminescence sandwich assay). Plasma albumin concentrations were analyzed using an automated clinical chemistry analyser (Randox Daytona Plus, NI). Serum concentrations of total calcium, triacylglycerol (TAG), and non‐esterified fatty acids (NEFA) were analyzed using an automated clinical chemistry analyser (Randox Daytona Plus, NI). Serum C‐reactive protein (CRP), and interleukin‐6 (IL‐6), leptin, and insulin, were measured using multiplex assays (V‐plex and U‐plex, respectively) on a QuickPlex SQ120 (Mesoscale Diagnostics, USA).

### Method Details—Adipose Tissue RNA Sequencing

Total RNA was extracted using RNeasy Mini Kit (Qiagen, UK) according to the manufacturer's instructions. Total RNA was quantified using RiboGreen (Invitrogen, USA) on the FLUOstar OPTIMA plate reader (BMG Labtech GmbH, UK), and the 4200 TapeStation (Agilent, RNA ScreenTape; Agilent Technologies, USA) used to measure the size profile and integrity of RNA. Ribosomal integrity number (RIN) estimates for all samples were between 3.0 and 8.9. RNA sequencing was performed on polyA‐enriched total RNA, on a NovaSeq6000 (Illumina, Inc., CA, USA) by the Wellcome Trust Oxford Genomics Centre (Oxford, UK).

Input material was normalized to 200 ng prior to library preparation. Polyadenylated transcript enrichment and strand‐specific library preparation were completed using NEBNext Ultra II mRNA kit (New England Biolabs Inc., USA) following the manufacturer's instructions. Libraries were amplified (11 cycles) on a Tetrad (Bio‐Rad Laboratories, CA, USA) using in‐house unique dual indexing primers.^[^ ^75^ ^]^ Individual libraries were normalized using Qubit, and the size profile was analyzed on the 2200 or 4200 TapeStation. Individual libraries were normalized and pooled together accordingly. The pooled library was diluted to ≈10 nmol L^−1^, denatured, and further diluted prior to loading on the sequencer. Paired end sequencing was performed using a HiSeq4000 75 bp platform (Illumina, HiSeq 3000/4000 PE Cluster Kit and 150 cycle SBS Kit), FastQ sequencing files were processed using the Galaxy web platform (usegalaxy.org), using the GRCh38/hg38 reference genome. Transcriptome wide false discovery rate (FDR) adjustments were applied, with an adjusted significance threshold of *q* < 0.05. Functional annotation was performed in the database for annotation, visualization, and integrated discovery (DAVID) 6.8 (2019 release^[^ ^76^, ^77^ ^]^) and Genesis 1.8.1.^[^ ^78^ ^]^ Pathway analysis was performed using Kyoto encyclopedia of genes and genomes (KEGG) and gene ontology (GO) terms, using a modified Fisher exact test (EASE (expression analysis systematic explorer)^[^ ^79^ ^]^) with a significance threshold of *P* ≤ 0.01.

The AmiGO web‐based gene ontology toolset was used to identify genes involved in vitamin D metabolism. Specifically, individual genes under GO terms: “vitamin D biosynthetic process,” “vitamin D catabolic process,” “vitamin D 23‐hydroxylase activity,” “vitamin D 25‐hydroxylase activity,” “vitamin D response element binding,” “vitamin D 24‐hydroxylase activity,” “vitamin D receptor signaling pathway,” “positive regulation of vitamin D biosynthetic process,” “regulation of vitamin D biosynthetic process,” “regulation of vitamin D 24‐hydroxylase activity,” “negative regulation of vitamin D biosynthetic process,” “regulation of vitamin D receptor signaling pathway,” “positive regulation of vitamin D 24‐hydroxylase activity,” “negative regulation of vitamin D receptor signaling pathway,” and “positive regulation of vitamin D receptor signaling pathway” were identified.

### Calculations

Total 25(OH)D was calculated as 25(OH)D_2_ plus 25(OH)D_3_.

Free 25(OH)D was calculated using the equation of Bikle et al.^[^ ^80^ ^]^ (see below), where Free 25(OH)D = concentration of free 25(OH) vitamin D in mol/L; *K*_alb_ = affinity constant between 25(OH) vitamin D and albumin in mol/L; *K*_DBP_ = affinity constant between 25(OH)D vitamin D and DBP in mol/L; albumin = concentration of total serum albumin in mol/L; DBP = concentration of total vitamin D‐binding protein in mol/L (assuming a MW of 58 000 g mole^−1^); Total 25(OH) vitamin D = concentration of total 25(OHD in mol/L).

(1) $$\mathrm{Free}\;25\left(OH\right)D=\frac{\mathrm{total}\;25\left(OH\right)D}{1+\left(K_{alb}\times \mathrm{albumin}\right)+\left(K_{DBP}\times DBP\right)}$$

Binding affinity constants used for 25(OH)D were:

(2) $$K_{\mathrm{DBP}}\cdot =\cdot 7\times 10^{8}$$

(3) $$K_{\mathrm{alb}}\cdot =\cdot 6\times 10^{5}$$

Bioavailable 25(OH)D was calculated using the equation below, where Free25(OH)D  =  concentration of free 25(OH)D in mol/L; *K*_alb_  =  affinity constant between 25(OH)D and albumin in mol/L; albumin  =  concentration of total serum albumin in mol/L.

(4) $$\mathrm{Bioavailable}\;25\left(\mathrm{OH}\right)D\;=\left(K_{\mathrm{alb}}\times \mathrm{albumin}\right)+1)\times \;\mathrm{Free}\;25\left(\mathrm{OH}\right)D$$

As a biomarker for whole body 25(OH)D3 expenditure, disappearance rate of d_3_‐25(OH)D_3_ stable isotope tracer in plasma was used to estimated half‐life using the line of best fit of the natural log of d_3_‐25(OH)D_3_ concentration against time.^[^ ^74^ ^]^

Adipose tissue insulin resistance index (Adipo‐IR; pmol/L × nmol/L) was calculated by multiplying fasting NEFA concentration (mmol/L) by fasting insulin concentration (pmol/L).

### Quantification and Statistical Analysis—Sample Size Estimation

Sample size calculations were based on our preliminary data of the effect of a 24‐week exercise intervention on circulating 25(OH)D during winter (Cohen's *d* = 0.95 for the difference in serum 25(OH)D concentration from *n* = 6 (exercise) and *n* = 5 (control) of participants taking part in this aforementioned study from October to March only^[^ ^81^ ^]^). With 80% power and an alpha level of 0.05, a sample size of 40 was estimated to be required to establish the effect of exercise compared to control in the present RCT (Cohen's *d* = 0.95). This was increased to *n* = 50 (25:25) to allow for dropout of 20% (drop out was ≈17% from our previous longer exercise intervention studies^[^ ^81^ ^]^).

### Quantification and Statistical Analysis—Randomization

Participants were randomized 1:1 to control and exercise groups in the first winter of testing, and then 1:2 to control and exercise groups in the second and third winters of testing. Group allocation was by minimization on the following baseline characteristics; sex, age, FMI, PAL, and Fitzpatrick Skin Phototype.^[^ ^23^ ^]^ A University of Bath member of staff not involved in the research conducted the randomization based on data collected during screening. Neither the participants nor the study investigators were blinded for group allocation.

### Quantification and Statistical Analysis—Statistical Analyses

Statistical tests are listed in figure legends, along with specific sample size for a given variable if it differs from control group *n* = 20 and exercise group *n* = 21.

A LMEM was used to examine the effect of the intervention on outcomes that might be expected to change with time over the intervention period in the control group, i.e., vitamin D metabolite concentrations, half‐life, and change in expression of selected genes associated with vitamin D metabolism that were expressed in adipose tissue. As such, effects of time, and group × time interaction effects are reported for these outcomes. The mean difference of the change scores and 95% confidence intervals were calculated, with Cohen's d effect size with 5000 sample bootstrapped 95% confidence intervals where relevant.^[^ ^27^ ^]^

For outcomes that would be expected to remain stable over time in the control group, a general linear model comparing change scores between groups with baseline as a covariate was employed. Group x time interaction effect, estimated mean difference, and 95% confidence intervals, and Cohen's *d* effect size with 5000 sample bootstrapped 95% confidence interval^[^ ^27^ ^]^ are presented for these outcomes to demonstrate effect of the intervention.

Correlations between “baseline” and absolute change in vitamin D metabolite concentrations were assessed with Pearson's correlation analysis for each group independently, using the Oldham method. To reduce the effect of mathematical coupling and avoid the effect of regression to the mean, the mean of baseline and post‐intervention was taken instead of baseline.^[^ ^28^, ^29^ ^]^ Differences in correlations between groups were compared using Fisher's *z* test and Zou's calculation of 95% confidence intervals.^[^ ^30^ ^]^

Given the sample size (> *n* = 30), normality of the residuals from the LMEM and ANCOVA analysis was considered by visual inspection of the Q–Q plots.^[^ ^26^ ^]^ Adjustment for multiple comparisons due to secondary outcomes was not undertaken as when secondary outcomes are interpreted precisely and exclusively, per‐comparison‐wise error rate is not increased.^[^ ^82^ ^]^

Data are presented as mean ± standard deviation unless stated otherwise. Statistical significance was accepted at *p* < 0.05. Effect sizes were calculated with *d* > 0.5 considered to be a moderate effect and *d* > 0.80 considered a large effect.^[^ ^83^ ^]^ Analysis was performed using GraphPad Prism 10.2.2 for Windows (GraphPad Software, Boston, MA), SPSS Statistics for Windows 28.0.0 (IBM Corp. Armonk, NY), and RStudio 2023.06.2 for Windows R. (PBC, Boston, MA) using the DABEST‐0.1.0 code for estimation statistics^[^ ^27^ ^]^ and cocor 1.1‐4 code fir comparison of correlations.^[^ ^30^ ^]^

### Quantification and Statistical Analysis—Clinical Trial Registration

The trial was registered on the ISRCTN registry (https://www.isrctn.com/ISRCTN29195046) and the full protocol is available through the registry.

## Conflict of Interest

The authors declare no conflict of interest.

## Author Contributions

Conceptualization: D.T., J.A.B., J.T.G., M.H., K.J., A.K., and O.J.P.; Data curation: O.J.P.; Formal analysis; O.J.P., S.E.D., C.J., M.A.L., S.R.M., and D.P.; Funding acquisition: D.T., J.A.B., J.T.G., M.H., K.J., and A.K.; Investigation: O.J.P. and S.E.D.; Methodology: O.J.P., D.T., M.H., K.J., J.G.T., and J.A.B.; Project administration: O.J.P. and D.T.; Resources: D.T., M.H., M.A.L., and K.G.; Supervision: D.T.; Validation: O.J.P., S.E.D., C.J., S.R.M., and D.A.P.; Visualization: O.J.P.; Writing – original draft: O.J.P. and D.T.; and Writing – review & editing: all authors.

## Data Availability

All other data needed to evaluate the conclusions in the paper are available through the University of Bath Data Archive (https://doi.org/10.15125/BATH‐01420), that is, all data reported in the present study at an anonymized participant level. These data will remain in the Data Archive indefinitely. Any additional information required to reanalyze the data reported in this paper is available from the corresponding authors upon request, and transfer agreements will be established as required.
