# Correction to “Exercise Without Weight Loss Prevents Seasonal Decline in Vitamin D Metabolites: The VitaDEx Randomized Controlled Trial”

**DOI:** 10.1002/advs.74595

**Published:** 2026-03-23

Perkin OJ^1^, Davies SE^1^, Hewison M^2^, Jones KS^3^, Gonzalez JT^1^, Betts JA^1^, Jenkinson C^4,5^, Lindsay MA^6^, Meadows SR^3^, Parkington DA^3^, Koulman A^3^, Thompson D^1^. Exercise Without Weight Loss Prevents Seasonal Decline in Vitamin D Metabolites: The VitaDEx Randomized Controlled Trial. Adv Sci (Weinh). 2025 Jun;12(22):e2416312. doi: 10.1002/advs.202416312. Epub 2025 May 11. PMID: 40349161; PMCID: PMC12165100.

^1^Centre for Nutrition and Exercise Metabolism, Department for Health, University of Bath, Bath, UK

^2^Institute of Metabolism and Systems Research, University of Birmingham, Birmingham, UK

^3^Nutritional Biomarker Laboratory, MRC Epidemiology Unit, University of Cambridge, Cambridge, UK

^4^University of Sydney, Sydney, New South Wales, Australia

^5^MRC Laboratory of Medical Sciences, Du Cane Road, London, UK

^6^Department of Life Sciences, University of Bath, Bath, UK

An error was made in the code used to analyze the exploratory gene expression data included in Figure 4. Where we believed we were reporting analysis of log2 counts per million data, we were in fact reporting the raw counts per million data.

The patterns of the data in Figure 4 are not markedly different, and this does not affect the overall findings, though the y‐axis on Figure 4c requires adjustment.

See below for amended y‐axis values:

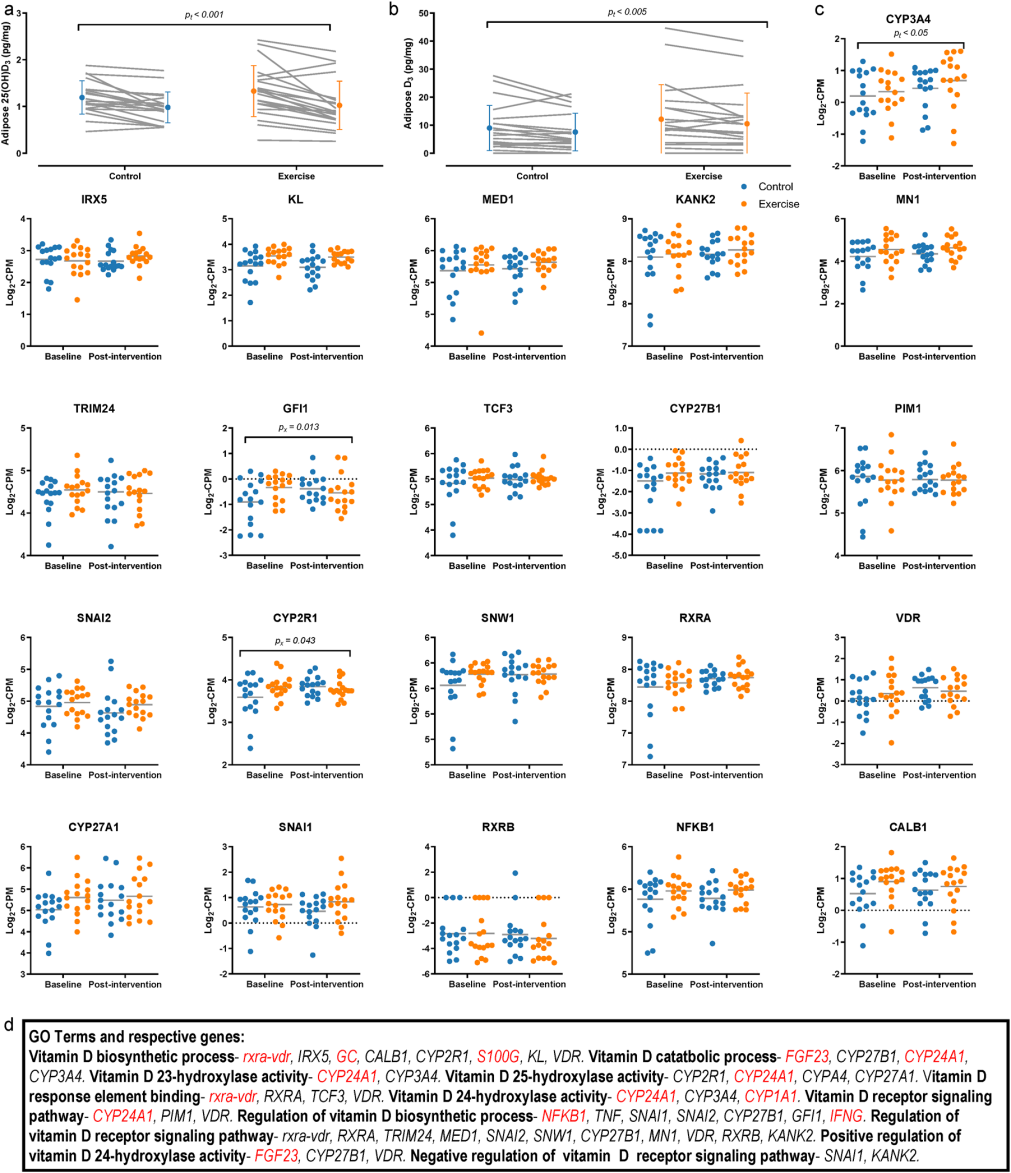

The inferences from these specific data do not change greatly; two genes that initially were close to showing a statistically significant interaction effect with unadjusted linear mixed effects modelling now do show a significant interaction effect (GFI1; *p_x_ = 0.013*, and CYP2R1; *p_x_ = 0.043*). However, in the discussion we had initially speculated on what an interaction effect in CYP2R1 might mean from a local vitamin D metabolism perspective, so we have amended this text accordingly. See below for the original discussion paragraph text

*It should also be highlighted that, if vitamin D metabolism occurs within adipose tissue [59] as the observed adipose expression of CYP27B1 and CYP2R1 suggests, then making inferences regarding vitamin D release from adipose based purely on metabolite concentration may be an oversimplification*.

*Original text: For example, a p‐value of 0.052 for an interaction effect in CYP2R1 expression in adipose between groups over the intervention period was observed*.

*Amended text: For example, p‐values of 0.013* and *0.043 for an interaction effect in GFI1* and *CYP2R1 expression in adipose between groups over the intervention period was observed.*

*If this led to a greater local 25‐ hydroxylase activity in the control group, then increased vitamin D3 conversion to 25(OH)D within adipose tissue could be expected. The fate of locally produced 25(OH)D is unclear, but this could mean that changes in adipose tissue concentrations of each metabolite may therefore reflect the combination of release of metabolites and their local conversion to other metabolites*.

We apologize for this error.

